# Adverse outcomes for chronic myeloid leukemia patients with splenomegaly and low in vivo kinase inhibition on imatinib

**DOI:** 10.1038/s41408-023-00917-4

**Published:** 2023-09-11

**Authors:** Chung H. Kok, Verity A. Saunders, Phuong Dang, Naranie Shanmuganathan, Deborah White, Susan Branford, David Yeung, Timothy P. Hughes

**Affiliations:** 1 Precision Cancer Medicine Theme, South Australian Health & Medical Research Institute (SAHMRI), Adelaide, SA Australia; 2 Adelaide Medical School, University of Adelaide, Adelaide, SA Australia; 3 Centre for Cancer Biology, SA Pathology, Adelaide, SA Australia; 4 Clinical Health Sciences, University of South Australia, Adelaide, SA Australia; 5 Department of Haematology, Royal Adelaide Hospital and SA Pathology, Adelaide, SA Australia; 6 Department of Genetics and Molecular Pathology, SA Pathology, Adelaide, SA Australia; 7 Australasian Leukaemia and Lymphoma Group (ALLG), Richmond, VIC Australia

**Keywords:** Chronic myeloid leukaemia, Translational research

## Abstract

Variability in the molecular response to frontline tyrosine kinase inhibitor (TKI) therapy in chronic myeloid leukemia may be partially driven by differences in the level of kinase inhibition induced. We measured in vivo BCR::ABL1 kinase inhibition (IVKI) in circulating mononuclear cells after 7 days of therapy. In 173 patients on imatinib 600 mg/day, 23% had low IVKI (<11% reduction in kinase activity from baseline); this was associated with higher rates of early molecular response (EMR) failure; lower rates of major molecular response (MMR), and MR4.5 by 36 months, compared to high IVKI patients. Low IVKI was more common (39%) in patients with large spleens (≥10 cm by palpation). Notably 55% of patients with large spleens and low IVKI experienced EMR failure whereas the EMR failure rate in patients with large spleens and high IVKI was only 12% (*p* = 0.014). Furthermore, patients with large spleen and low IVKI had a higher incidence of blast crisis, inferior MMR, MR4.5, and event-free survival compared to patients with large spleen and high IVKI and remaining patients. In nilotinib-treated patients (*n* = 73), only 4% had low IVKI. The combination of low IVKI and large spleen is associated with markedly inferior outcomes and interventions in this setting warrant further studies.

## Introduction

Chronic myeloid leukemia (CML), caused by constitutively active BCR::ABL1 fusion tyrosine kinase, has served as a paradigm for the successful application of molecularly targeted cancer therapy. Tyrosine kinase inhibitors (TKIs) have transformed the lives of CML patients, with excellent long term outcomes in responding patients [1–5]. Patients who receive second generation TKIs as frontline therapy achieve faster and deeper molecular responses (MRs) than those treated with imatinib, presumably due to their greater potency [6]. However, the level of kinase inhibition actually achieved on first or second generation TKIs has not been systematically assessed or compared. To date, even with the current choice of four frontline TKIs (imatinib, nilotinib, dasatinib, and bosutinib), 15–20% of CML patients respond poorly and around half of these poor responders will die due to CML progression or complications post allograft [7].

Moreover, studies have shown that patients with a longer *BCR::ABL1*(IS) halving time corresponding to a slower initial *BCR::ABL1*% decline have an inferior clinical outcome, inferior MR, and higher probability of relapse in the setting of an attempt at treatment-free remission (TFR) [8–10].

Crkl (Crk-like protein) is an immediate downstream substrate for the BCR::ABL1 protein. Phosphorylation of Crkl occurs as a direct consequence of BCR::ABL1 kinase activity, with levels of phosphorylated Crkl (pCrkl) compared to total Crkl protein providing the most practical surrogate measure of kinase activity of BCR::ABL1 [11, 12]. It has been shown that measuring the ratio of pCrkl to Crkl in vitro using mononuclear cells is predictive of cytogenetic and MR by 12 months [13, 14]. We have previously shown, based on an in vivo kinase inhibition (IVKI) assay developed in-house, that greater than 50% kinase inhibition during the first month of TKI therapy is associated with a high probability of MMR at 12 months in patients treated with imatinib [15]. We hypothesized that the IVKI measured as early as 7 days would correlate with clinical outcome and hence may prove to be a clinically relevant way to assess the adequacy of therapy and the potential value of early TKI dose adjustment or switching. In this study, we investigated the relationship between IVKI in patients after the first week of TKI treatment and subsequent MR.

## Materials and methods

### Patient samples

Blood samples at diagnosis (baseline, pre-TKI therapy) and after the first 7 days of treatment were collected for IVKI assessment from adult chronic phase CML (CP-CML) patients enrolled in the Australian TIDEL-II study [16] (ACTRN12607000325404) and the Evaluating Nilotinib Efficacy and Safety in Clinical Trials-Extending MRs (ENESTxtnd, NCT01254188) study [17]. Briefly, in the TIDEL-II study, CP-CML patients were started on 600 mg daily of imatinib. Failure to achieve time-dependent molecular milestones (consistent with optimal targets established in 2013 by the European LeukemiaNet [18]) led to either an increase in imatinib dose to 800 mg daily or a switch to nilotinib 400 mg twice daily (BID) [16]. Patients were also switched to nilotinib for imatinib intolerance [16]. In the ENESTxtnd study, CP-CML patients were started on 300 mg of nilotinib BID [17].

### In vivo kinase inhibition measurements

The percentage of kinase activity was assessed as previously described [15] and defined as the ratio of pCrkl to total Crkl x 100%. Kinase inhibition (IVKI) was defined as the percent reduction in kinase activity from day 0 to day 7.

### Statistical analysis

MR rates were calculated by cumulative incidence and compared using the Fine and Gray [19] test as previously described [20]. An event was defined as achievement of the MR of interest, either major MR (MMR, *BCR::ABL1* ≤ 0.1% IS) or MR4.5 (≤0.0032% IS) at 2 consecutive time points. Competing risks included permanent discontinuation of TKI treatment for any reason (including death).

Survival analyses, including event-free survival (EFS) and failure-free survival (FFS), were performed using the Kaplan–Meier method, and comparisons were made using the log-rank test. An event in EFS was defined as progression to accelerated phase or blast crisis, loss of either MMR or complete cytogenetic response, acquisition of *BCR::ABL1* mutations, death, or failure to achieve targets set by the European LeukemiaNet [18] in 2013 (*BCR::ABL1* > 10% IS at 6 months or *BCR::ABL1* > 1% IS at 12 months). FFS included all the EFS events as well as discontinuation of imatinib or nilotinib for any reason. Two-group comparison was performed using the Mann–Whitney *U* test or Student’s *t* test for continuous variables and Fisher’s exact test for categorical variables. Two-sample test for equality of proportions was performed using 2-sample proportion test. The threshold for the in vivo inhibition level of kinase was determined using the decision tree algorithm and the maximum sum of the sensitivity and specificity metrics implemented in the rpart [21] and cutpointr [22] R packages respectively. Analysis and graphical plots were performed using GraphPad Prism 9 (GraphPad Software Inc., La Jolla CA) or R statistical software v4.1.1. This is detailed further in supplementary methods.

## Results

### In vivo kinase inhibition by imatinib

**Fig. 1:**
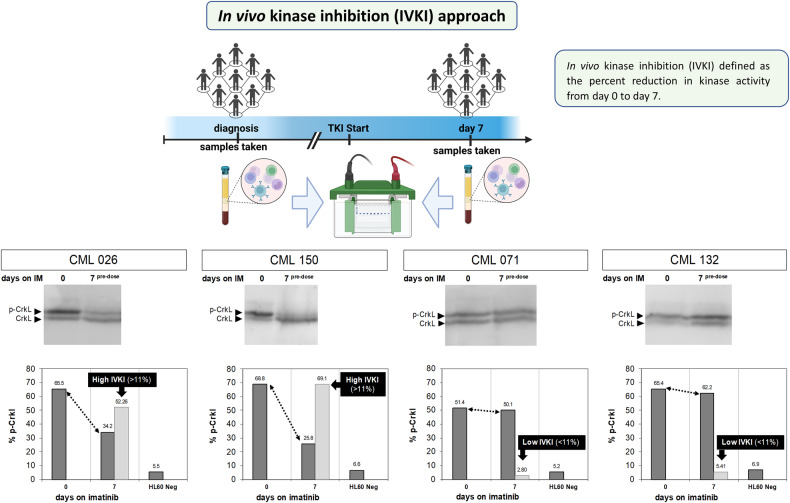
Schematic workflow of in vivo kinase inhibition (IVKI) assay. Anti-Crkl western blotting of PB-MNC total protein lysates prior to imatinib (IM) start versus 7 days on IM (pre-dose collection = trough IM plasma level) were performed one patient per SDS-PAGE gel. A lysate from BCR::ABL1 negative cell-line HL-60 (Neg Ctrl) was run per gel (not shown). Densitometric analysis graphs are shown below the western blot images. Actual %p-Crkl (representing Kinase Activity) is shown in dark gray bars; Light gray bars represent in vivo kinase inhibition (IVKI) at day 7 of IM therapy relative to untreated, assessed with respect to the no-p-Crkl negative control. (CML 026 and CML 150) patients exhibiting High IVKI > 11% at day 7. (CML 071 and CML 132) patients exhibiting Low IVKI < 11% at day 7. Upper panel figure was created with BioRender.com (agreement number: ZI25QYDCFT).

**Fig. 2:**
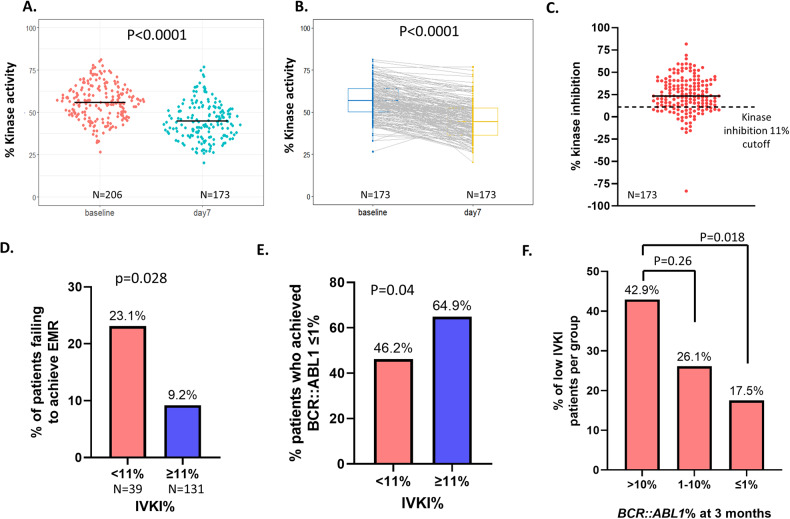
The heterogeneous level of in vivo p-Crkl kinase inhibition and its association with *BCR::ABL1*% level at 3 months. **A** Dotplot shows in vivo kinase activity (%p-Crkl) at baseline and at day 7 since imatinib treatment. The horizontal bar indicates median. **B** Boxplot and pairwise lineplot shows the dynamic of in vivo kinase activity for each patient during the first 7 days of imatinib treatment. **C** Dotplot shows the in vivo kinase inhibition (IVKI) for each patient after the first 7 days of imatinib treatment. The horizontal bar indicates median. The horizontal dotted line indicates IVKI threshold cutoff. **D** Barplot shows the proportion of EMR failure patients in low and high IVKI patient group. **E** Barplot shows the proportion of patients who achieved *BCR::ABL1* ≤ 1% at 3 months in low and high IVKI patient group. **F** Barplot shows the proportion of low IVKI patients in each *BCR::ABL1%* level at 3 months.

We assessed in vivo kinase activity at one or more timepoints for 206 CP-CML patients enrolled in the TIDEL-II study. The schematic workflow and examples of the pCrkl assay to determine in vivo kinase inhibition (IVKI) are shown in Fig. 1. Of these 206 patients, 173 had in vivo kinase activity data measured at both baseline and day 7. The demographic data for these patients is shown in Table 1. The kinase activity was significantly lower in patients tested after 7 days of imatinib compared to pre-TKI patients (45% vs 56%, *p* < 0.0001, Fig. 2A, B). The median level of IVKI was 23.3% (Fig. 2C).

**Table 1:** Patient characteristics in imatinib treated (TIDEL-II) and nilotinib treated (ENESTxtnd) cohorts.

Variable	Level	imatinib cohort	nilotinib cohort
*n*		173	73
Age, years (mean (SD))		49.32 (16.03)	50.57 (16.25)
*BCR::ABL1* halving time, days (mean (SD))		17.63 (12.80)	12.57 (10.76)
Gender (%)	Male	102 (59.0)	42 (57.5)
	Female	71 (41.0)	30 (41.1)
	N/A	0 (0)	1 (1.4)
*BCR::ABL1*% level at 3 months (%)	>10%	21 (12.1)	1 (1.4)
	1–10%	46 (26.6)	10 (13.7)
	≤1%	103 (59.5)	54 (74.0)
	N/A	3 (1.7)	8 (11.0)
*BCR::ABL1 t*ranscript type (%)	e13a2	72 (41.6)	24 (32.9)
	e14a2	63 (36.4)	35 (47.9)
	e13a2 & e14a2	36 (20.8)	14 (19.2)
	e13a3	1 (0.6)	0 (0)
	e1a2	1 (0.6)	0 (0)
*BCR::ABL1* halving time quartile (%)	Q1	31 (17.9)	24 (32.9)
	Q2	57 (32.9)	30 (41.1)
	Q3	44 (25.4)	7 (9.6)
	Q4	34 (19.7)	3 (4.1)
	N/A	7 (4.0)	9 (12.3)

*N/A* not available.

### Patients with low IVKI were significantly associated with EMR failure

Early molecular response (EMR) failure (*BCR::ABL1* ≥ 10% at 3 months) is known to correlate with inferior clinical outcome and long-term MRs in CML [23, 24]. In the frontline imatinib treated (TIDEL-II) [16] cohort, 12% of imatinib-treated patients did not achieve EMR. We determined an optimal IVKI threshold to predict EMR failure to be 11%, using two independent statistical approaches (Supplementary Fig. 1). Interestingly, there was no evidence of improvement in MRs and halving time for patients with higher IVKI, as long as they were above the 11% IVKI threshold (Supplementary Fig. 2). We defined patients with IVKI < 11% as low IVKI, and IVKI ≥ 11% as high IVKI. Almost a quarter (~23%) of imatinib treated patients had low IVKI (*n* = 39).

Patients with low IVKI were more likely to fail to achieve EMR compared to patients with high IVKI (23% vs 9%, OR:2.98, 95% CI:1.21–7.61, *p* = 0.028, Fig. 2D). Consistent with this, a significantly higher proportion of high IVKI patients achieved *BCR::ABL1* ≤ 1% at 3 months, as compared with low IVKI patients (64.9% vs 46.2%, *p* = 0.04, Fig. 2E). As shown in Fig. 2F, 43% of patients who failed EMR had low IVKI compared to 18% of patients who were *BCR::ABL1* ≤ 1% at 3 months (*p* = 0.018).

### Low IVKI level was associated with worse molecular response

Next, we determined whether low IVKI could predict inferior rates of MMR and MR4.5 in the imatinib-treated patient cohort. Based on ELN recommendations, the optimal response at 12 months is the achievement of MMR. Patients with low IVKI had a significantly lower MMR rate (46% and 69%) compared to patients with high IVKI (65% and 82%) by 12 (*p* = 0.01) and 24 months (*p* = 0.026) respectively (Supplementary Fig. 3A). The difference in MMR rate persisted at 36 months: 72% in low vs 85% in high IVKI patients respectively (*p* = 0.027, Supplementary Fig. 3A).

Low IVKI patients also had an inferior achievement of deep MR MR4.5 compared to high IVKI patients by 36 months (26% v 44%; *p* = 0.037, Supplementary Fig. 3B). Patients with low IVKI had a numerically higher rate of transformation to blast crisis than patients with high IVKI, but this did not reach statistical significance (7.7% vs 3%, OR 2.71, 95% CI 0.65–10.39, *p* = 0.19, Supplementary Fig. 3C). There was no statistical difference in *BCR::ABL1* kinase domain mutation development when comparing low and high IVKI patients (5% vs 6%, *p* = 1.0, Supplementary Fig. 3D).

There were no statistically significant differences in EFS (81% vs 81%, *p* = 0.97) and FFS (71% vs 74%, *p* = 0.72) when comparing patients with low IVKI and high IVKI (Supplementary Fig. 4). In this TIDEL-II imatinib treated cohort, 35% (*n* = 61) patients were switched to nilotinib for imatinib resistance or intolerance [16]. We determined whether there was a difference in the probability of meeting the trial-defined criteria for a switch to nilotinib between low and high IVKI patient groups. There was a significantly higher probability of low IVKI patients switching to nilotinib due to resistance compared to high IVKI patients (40% vs 18%, *p* = 0.002, Supplementary Fig. 5A). In contrast, there was no difference in nilotinib switch due to intolerance between low and high IVKI patient groups (15% vs 17%, *p* = 0.9, Supplementary Fig. 5B).

### Combination of large spleen and low in vivo kinase inhibition is an early predictor of inferior molecular response and poor outcome

**Fig. 3:**
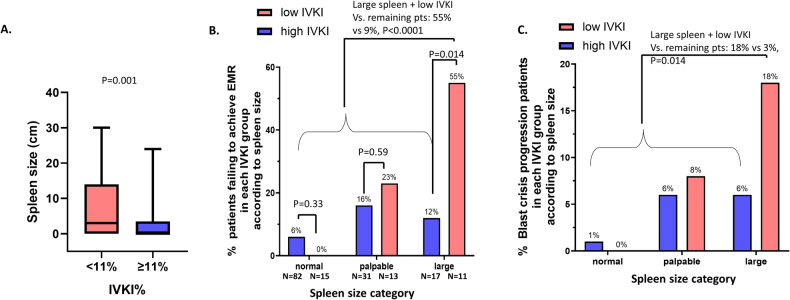
Combination of large spleen and low in vivo kinase inhibition is an early predictor of inferior molecular response and poor outcomes. **A** Boxplot shows the spleen size between low (<11%) and high (≥11%) IVKI patient groups. **B** Barplot shows the percentage of EMR failure patients in each IVKI group according to the spleen size groups. Red color indicates patients with low IVKI, and blue color indicates patients with high IVKI. **C** Barplot shows the percentage of blast crisis progression patients according to the combination of spleen size and IVKI groups.

Among baseline clinical characteristics examined (Table 2), only spleen size was different comparing low IVKI patients to high IVKI patients (3 cm vs 0 cm, *p* = 0.01) (Fig. 3A and Supplementary Fig. 6A). Patients with high EUTOS long-term survival (ELTS) score have inferior survival and MRs compared to low ELTS score [7, 25–27]. Spleen size is one of a parameter for calculating ELTS score. As expected, there was a significantly lower level of IVKI in patients with a high ELTS score (median 14%) than in patients with a low (26%, *p* = 0.01) and intermediate ELTS score (28%, *p* = 0.008, Supplementary Fig. 6B). We next divided patients into 3 categories according to spleen size; no palpable spleen (0 cm), palpable spleen (1–9 cm) and large spleen (≥10 cm). EMR failure rates increased according to spleen category (*p* = 0.001, Supplementary Fig. 6C). 17% of imatinib-treated patients had a large spleen and 39% of them had low IVKI. Notably 55% of patients with large spleen and low IVKI experienced EMR failure whereas the EMR rate in patients with large spleen and high IVKI was only 12% (*p* = 0.014, Fig. 3B). Moreover, we identified low IVKI and large spleen outperformed current prognostic scoring systems (Sokal [7, 28], ELTS [29], EUTOS [30]) that included spleen size to predict EMR failure (Supplementary Table 1). By multivariate analysis, low IVKI and large spleen (*p* = 0.003) was significant after adjusting for age, platelets count, blasts percentage, gender, transcript type and imatinib plasma trough level on day 7 (Supplementary Table 2). In addition, low IVKI and large spleen (*p* < 0.01) was significant after adjusting for Sokal or ELTS or EUTOS (Supplementary Table 2).

**Table 2:** Baseline clinical characteristics of imatinib treated patients with low or high IVKI.

Variable	low IVKI	high IVKI	*p*-value	BH adjusted *p*-value
*n*	39	134		
Age, years (median [IQR])	47.00 [37.50, 61.00]	50.00 [39.00, 63.00]	0.417	1.000
Gender (%) Male / Female	25 (64.1)/14 (35.9)	77 (57.5)/57 (42.5)	0.579	1.000
Baseline *BCR::ABL1* (IS) transcript % (median [IQR])	85.45 [42.20, 146.25]	84.20 [45.19, 141.50]	0.636	1.000
Baseline BCR::ABL1 kinase activity % (median [IQR])	54.72 [50.50, 63.56]	57.34 [50.06, 64.60]	0.373	1.000
WCC x10^9^/L (median [IQR])	43.00 [15.50, 95.25]	38.05 [17.28, 62.40]	0.28	1.000
Spleen Size, cm (median [IQR])	3.00 [0.00, 14.00]	0.00 [0.00, 3.00]	0.001	0.014
Platelets x10^9^/L (median [IQR])	310.50 [231.50, 432.50]	378.00 [271.00, 616.00]	0.036	0.432
Blasts% (median [IQR])	0.00 [0.00, 2.00]	0.00 [0.00, 1.00]	0.508	1.000
Neutophils% (median [IQR])	67.99 [58.42, 73.76]	64.44 [58.12, 72.77]	0.762	1.000
Bands% (median [IQR])	0.00 [0.00, 7.14]	0.00 [0.00, 5.00]	0.521	1.000
Monocytes% (median [IQR])	4.50 [2.89, 7.35]	3.98 [2.00, 6.04]	0.396	1.000
Lymphocytes% (median [IQR])	7.00 [4.00, 12.03]	10.00 [6.06, 16.77]	0.015	0.195
Basophils% (median [IQR])	2.99 [2.00, 5.71]	3.98 [1.79, 6.09]	0.568	1.000
Eosinophils% (median [IQR])	1.93 [0.96, 3.04]	2.01 [1.00, 3.05]	0.452	1.000

*WCC* white cell count, *IQR* interquartile range.

Patients with large spleen and low IVKI had higher incidence of blast crisis progression compared to the remaining patients (18% vs 3%, *p* = 0.014, Fig. 3C), but no difference in rate of mutation development (9% vs 6%, *p* = 0.63; Supplementary Fig. 6D).

We next divided into 3 groups: patients with large spleen and low IVKI, or large spleen with high IVKI, and the remaining patients to determine the relationship of these groups with MR and outcome. Patients with large spleen and low IVKI had higher incidence of failing EMR (55% vs 12% vs 9%, *p* = 0.0008; Supplementary Fig. 7A), and of blast crisis progression (18% vs 6% vs 3%, *p* = 0.04; Supplementary Fig. 7B), compared to patients with large spleen and high IVKI, and the remaining patients respectively. However, there was no statistical difference in the rate of *BCR::ABL1* kinase domain mutation between the groups (9% vs 12% vs 5%, *p* = 0.23; Supplementary Fig. 7C).

Patients with large spleen and low IVKI had inferior MMR (46% vs 77% vs 86%, *p* = 0.007; Supplementary Fig. 8A) and MR4.5 (0% vs 18% vs 45%, *p* = 0.0024; Supplementary Fig. 8B) compared to patients with large spleen and high IVKI, and the remaining patients respectively. Moreover, patients with large spleen and low IVKI had inferior EFS (60% vs 68% vs 84%, *p* = 0.04) and FFS (55% vs 64% vs 76%, *p* = 0.05) compared to patients with large spleen and high IVKI, and the remaining patients respectively (Supplementary Fig. 8C, D).

The Organic Cation Transporter 1 (OCT-1) is the main influx pump for imatinib [31]. Low OCT-1 activity (OA) which corresponds to low uptake of imatinib, has been associated with poor MR [32]. Although a higher proportion of patients with low OA was observed in the group with low IVKI compared to the high IVKI group, this difference did not achieve statistical significance (54% vs. 42%, *p* = 0.2, Supplementary Fig. 9A). Likewise, patients with large spleen size and low IVKI exhibited a higher proportion of low OA compared to patients with large spleen size and high IVKI and the remaining patients respectively (64% vs. 35% vs. 44%, *p* = 0.33, Supplementary Fig. 9B).

### Patients with low IVKI had longer halving times

**Fig. 4:**
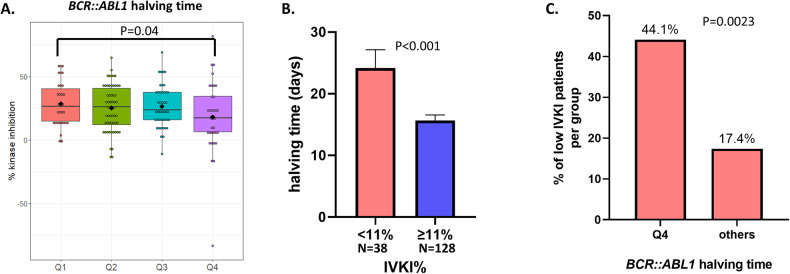
Low IVKI patients treated with imatinib have longer halving time. **A** Boxplot shows the IVKI level in each *BCR::ABL1* halving time quartile for patients treated with imatinib. For the halving time quartile, Q1 < 9.35 days, Q2: 9.35–13.95 days, Q3: 13.96-21.85 days, and Q4: >21.85 days. Diamond shape represents mean and horizontal line in each boxplot represents median. Each dot represents a patient sample. **B** Barplot shows the average of *BCR::ABL1* halving time in the low (<11%) and high (≥11%) IVKI patient groups. The error bar represents standard error measurement. **C** Barplot shows the proportion of low IVKI patients in each *BCR::ABL1* halving time groups. For the halving time quartile, others: ≤21.85 days, and Q4: >21.85 days.

To determine the relationship between *BCR::ABL1* mRNA halving time and IVKI, we divided halving time into quartiles as previously defined [10]. Quartile 4 (Q4) halving time comprises patients with the longest halving time corresponding to the slowest rate of *BCR::ABL1% IS* decline in the first 3 months of TKI treatment. Patients with a Q4 halving time had a significantly lower level of IVKI compared to patients with a Q1 halving time (median 17% vs 27%, *p* = 0.04, Fig. 4A). However, there were no statistically significant IVKI differences in patients with Q2 (26%, *p* = 0.1) or Q3 (24%, *p* = 0.07) halving time when compared to Q4 (17%).

Patients with low IVKI on average had longer halving time compared to higher IVKI patients (24 days vs 16 days, *p* < 0.001, Fig. 4B). In addition, 44% of Q4 halving time patients had low IVKI compared to 17% in patients from Q1-3 quartiles (*p* = 0.0023, Fig. 4C).

### No difference in imatinib plasma trough levels between low and high IVKI patient groups

Imatinib plasma trough levels were measured from the same blood sample used for IVKI assessment. Surprisingly there was no significant difference in imatinib plasma trough level between low and high IVKI patient groups on day 7 (1629 vs 1648 ng/mL, *p* = 0.74, Supplementary Fig. 10A, B). It has been shown previously that high imatinib plasma trough level (>1000 ng/mL, collected between 21 and 27 h after last drug administration) is associated with MMR achievement at 12 months [33]. However, there were no differences in IVKI level when patients with high and low imatinib plasma trough level at day 7 were compared (21% vs 24%, *p* = 0.24, Supplementary Fig. 10C). There was no significant difference in proportion of patients with low IVKI in low imatinib plasma trough level group when compared to high plasma trough level group (27% vs 22%, *p* = 0.51, Supplementary Fig. 10D).

### Nilotinib is more potent than imatinib in inhibiting BCR::ABL1 kinase activity in vivo

**Fig. 5:**
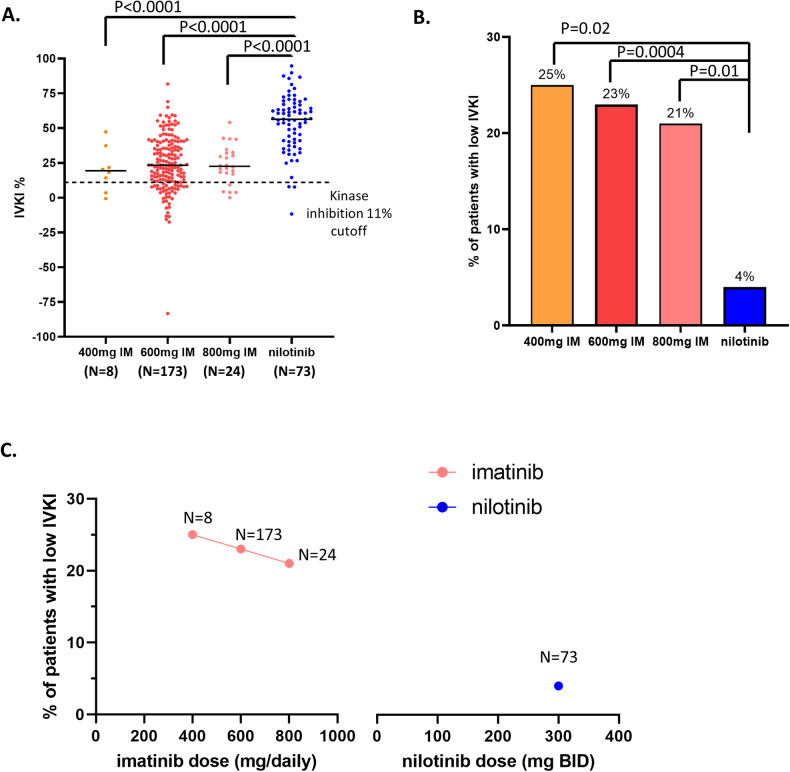
CML patients treated with nilotinib had higher in vivo kinase inhibition than those patients treated with imatinib. **A** Dotplot shows IVKI level in patients treated with 400 mg (*n* = 8), 600 mg (*n* = 173), 800 mg daily imatinib (*n* = 24) or 300 mg BID nilotinib (*n* = 73). The horizontal bar indicates median. The horizontal dotted line indicates IVKI threshold cutoff (11%). IM indicates imatinib. **B** Barplot shows the proportion of patients with low IVKI in each TKI treatment: 400 mg (*n* = 8), 600 mg (*n* = 173), 800 mg daily imatinib (*n* = 24) or 300 mg BID nilotinib (*n* = 73). **C** Dotplot shows the frequency of low IVKI patients in percentage among imatinib cohorts treated with different dose (400, 600, 800 mg daily) and nilotinib treated cohort (300 mg BID).

We performed in vivo kinase activity at both baseline and day 7 for 73 CP-CML patients enrolled in the ENESTxtnd study [17]. The demographic data for these patient samples are shown in Table 1. Patients treated with nilotinib had significantly higher IVKI compared to patients treated with imatinib (median 54% vs 19% (400 mg), 23% (600 mg) or 23% (800 mg), *p* < 0.0001, Fig. 5A). There were only 3/73 (4%) nilotinib treated patients with low IVKI (<11%) compared to 2/8 (25%), 39/173 (23%), 5/24 (21%) low IVKI patients treated with 400 mg (*p* = 0.02), 600 mg (*p* = 0.0004), and 800 mg (*p* = 0.01) imatinib respectively (Fig. 5B). Our analysis indicates a potential inverse relationship between the dosage of TKI and the frequency of low IVKI among patients, albeit with a limited interpretation due to the small sample size (Fig. 5C). Additionally, there was a higher proportion of Q1 halving time (38% vs 19%), and lower proportion of Q4 halving time (5% vs 21%) in patients treated with nilotinib compared to imatinib (*p* < 0.0001, Supplementary Fig. 11).

### The relationship between IVKI and superior response seen in nilotinib

There was only one patient who failed EMR in the nilotinib cohort (1.4%, Table 1) compared to 12% in the imatinib treated cohort. Thus, we used a MR of *BCR::ABL1* ≤ 1% at 3 months as the main response indicator for comparison. Nilotinib treated patients (ENESTxtnd) had higher IVKI and higher proportion of patients achieving superior MR at 3 months (*BCR::ABL1* ≤ 1%) compared to imatinib treated patients (TIDEL-II) (83% vs 61%, *p* = 0.001, Supplementary Fig. 12).

We determined whether patients receiving nilotinib have equivalent outcomes to imatinib treated patients after adjusting for IVKI. We hypothesized that there would be little or no difference in the proportion of patients with *BCR::ABL1* ≤ 1% at 3 months between imatinib and nilotinib, if the degree of kinase inhibition was equivalent. We performed propensity score matching with IVKI as a covariate to ensure the IVKI level was distributed equally between TKIs (Supplementary Fig. 13A–C and Supplementary Table 3). However, we observed a significant difference in the proportion of patients achieving *BCR::ABL1* ≤ 1% at 3 months between TKIs after adjusting for IVKI (nilotinib—88% vs imatinib – 67%, *p* = 0.04, Supplementary Fig. 13D). This suggested that superior MR observed in nilotinib compared to imatinib may be only partially driven by superior IVKI.

### No difference was identified between IVKI and molecular responses in patients treated with nilotinib

There were only 3 patients with low IVKI (<11%) in this nilotinib treated cohort, so we divided IVKI in this cohort into quartiles to determine the impact of IVKI quartile on MR by 24 months. There were no statistically significant differences between IVKI quartile groups with regards to the achievement of MMR (*p* = 0.99, Supplementary Fig. 14A) or MR4.5 (*p* = 0.42, Supplementary Fig. 14B) by 24 months.

### The relationship of IVKI and treatment-free remission (TFR)

The median follow-up for TIDEL-II cohort was 37 months (2.5–48 months) and ENESTxtnd cohort was 23.8 months (1.3–126.4 months). Of these 244 TIDEL-II and ENESTxtnd patients who were evaluated for IVKI, 24 patients have so far achieved eligibility for TFR and had molecular relapse outcome out to 12 months after stopping TKI. Of these 24 patients, 13 (54.2%) patients relapsed, defined by loss of MMR. For those who relapsed, the duration of MR4 prior to stopping was 3.5 years (range: 2.3–4.8) and MR4.5 was 2.9 years (2.1–4.8) compared to patients who sustained TFR where duration of MR4 prior to stopping was 4 years (2.8–5.33), and duration of MR4.5 was 2.9 years (1.9–5.33).

Moreover, of these 24 patients, four patients had low IVKI, and 20 patients had high IVKI. Low IVKI patients had 25% (*n* = 1/4) rate of TFR compared to 50% (*n* = 10/20) TFR rate for patients with high IVKI (OR: 3.7; 95% CI: 0.45–51.3).

## Discussion

We have demonstrated that the level of kinase inhibition achieved in vivo can be reliably measured ex vivo after 7 days of TKI therapy and, in the imatinib setting, has clinical significance. This study differs from our previously published study [15], based on a cohort of 49 patients, which was only able to identify a clinically significant level of IVKI by including days 7,14,21 and 28 assessment timepoints. In this earlier study we could not identify a level of IVKI that was associated with inferior outcomes, nor did we identify the critical importance of spleen size and its relevance to the interpretation of IVKI. The level of IVKI achieved with imatinib is presumably determined by a composite of variables including drug absorption, drug metabolism including drug-drug interactions, and the efficiency of OCT-1 transporter mediated influx and of ABCB1, and ABCC3 mediated efflux [32, 34–37]. The composition of cell types within the mononuclear cells collected may also be relevant. Rather than assessing all of these variables individually, IVKI provides a direct measure of the impact of imatinib on BCR:ABL1 kinase activity. Interestingly we observed no correlation between imatinib plasma trough level and IVKI, again highlighting the importance of IVKI as a summative measure. However, IVKI is technically challenging to perform, and can only be reliably measured while leukemic cells are still the predominant population in the blood, limiting its use to the initial days of treatment as most patients will have a rapid clearance of leukemic cells from peripheral circulation after a few weeks of TKI therapy [15].

Patients receiving frontline imatinib who had a low IVKI level on day 7 had significantly inferior MR with a higher risk of failing EMR, MMR, and a lower probability of achieving MR4.5, a prerequisite for consideration of TFR. Importantly, patients with low IVKI also had longer halving times, another indication that their prospects of achieving TFR would be relatively low. A significant proportion of patients, treated with 600 mg/day of imatinib, will have low IVKI (23% in TIDEL-II). Based on our findings, low IVKI is likely to be more common in the group receiving the standard dose (400 mg/day) of imatinib.

Patients with large spleens at diagnosis were more likely to have low IVKI at day 7 on imatinib. We speculate that this may be related to sequestering in the spleen of primitive leukemic progenitors and leukemic stem cells [38, 39] that may be less exposed to imatinib and capable of producing circulating myeloid progeny that are still relatively kinase active. From a clinical perspective, the most important finding was that patients with large spleens are substantially more likely to have low IVKI and that the prognostic significance of having a low IVKI was most pronounced in this subgroup. Many regions around the world tend to prioritize the use of second-generation TKIs for patients exhibiting an intermediate or high Sokal score [40, 41]. Nevertheless, our analysis reveals a noteworthy distinction: patients with both a large spleen and low IVKI levels exhibit poorer outcomes compared to patients with large spleen but high IVKI levels. These findings provide valuable guidance to clinicians in their selection of TKIs.

In contrast to imatinib treated patients, those receiving frontline nilotinib had much higher median IVKI levels with only 4% being in the low IVKI category, compared to 23% of patients receiving imatinib 600 mg daily. This in vivo data further supports the notion that nilotinib is more effective at targeting BCR::ABL1 kinase activity than higher dose imatinib, consistent with the finding that nilotinib leads to faster and deeper MRs [6, 7, 40]. It further suggests that IVKI is of limited relevance in the nilotinib setting because low IVKI is rarely observed. We also noted a threshold effect regarding the correlation between IVKI and MR: regardless of treatment received, once IVKI is above 11%, MR is not improved by achieving higher levels of IVKI. However, higher IVKI achieved on nilotinib does not appear to be sufficient to explain the superior MRs seen with nilotinib and is likely to only make a partial contribution to the differences observed between the two drugs.

One limitation of our study is that our current assay result is based on measuring kinase activity (ratio of pCrkl: total Crkl protein level) in a mixed cell population within the mononuclear cell fraction. It is possible that measurement of IVKI in immature myeloid cells could provide a more accurate surrogate measure of kinase inhibition in the most critical cell populations. Mononuclear cells were chosen for these studies because they are easily isolated, a critical consideration in a multicentre trial. With advanced technology, such as mass cytometry [42, 43] or flow cytometry, it is possible that IVKI could be measured in specific cell subsets, which might prove to be more clinically relevant. This approach may offer a more accurate reflection of kinase inhibition in pivotal cell populations. Translating this into practice could give further confidence for clinicians to continue treating most patients with an easily affordable drug with an excellent long term safety profile, while also providing an early indication that a more potent TKI may be needed in selected patients.

In conclusion, early assessment of the level of kinase inhibition achieved in patients receiving imatinib in the frontline setting has the potential to identify patients who are not gaining adequate suppression of BCR::ABL1 kinase activity. These patients are at higher risk of failing to achieve key molecular targets and subsequent poorer outcomes, including failing to achieve sustained deep MRs, and thus not reaching subsequent eligibility for TFR. In the subset of patients with large spleens a low IVKI is also associated with inferior EFS and FFS. The capacity to identify these patients as early as day 7 after the initiation of treatment opens a unique window of opportunity to optimize the TKI dose or consider switching to a more potent TKI well before time-dependent molecular targets are assessable. This is especially important for patients with large spleens. Our findings have particular relevance in low- and middle-income countries, where large spleens are more common on presentation. Available evidence suggests that the median spleen size at diagnosis is 9–15 cm across four low income countries [44–48]. In these communities imatinib is widely used, so the risk of adverse outcomes due to the combination of large spleen and low IVKI would apply to many patients.

Having established that this biomarker has clinical relevance in the imatinib setting, further work is indicated to refine this assay. The goal would be to provide a practical, accurate and standardized measure of kinase inhibition at an early timepoint to enable clinicians to adjust ongoing therapy for CML patients receiving frontline imatinib with a particular focus on patients with large spleens.

### Reporting summary

Further information on research design is available in the Nature Research Reporting Summary linked to this article.

### Supplementary information

Reporting Summary

Supplementary Materials

## Data Availability

The data supporting the findings of this study can be obtained from the corresponding author upon a reasonable request.
