# Supplementary material for: Adverse outcomes for chronic myeloid leukemia patients with splenomegaly and low in vivo kinase inhibition on imatinib
Source: Blood Cancer J. 2023 Sep 11;13(1):143. doi: 10.1038/s41408-023-00917-4 (PMC10495334; doi:10.1038/s41408-023-00917-4)
Supplement: Supplementary file 2 — Supplementary Materials [file 41408_2023_917_MOESM2_ESM.pdf]

Supplementary Materials

Adverse outcomes for chronic myeloid leukemia patients with splenomegaly and low *in vivo* kinase inhibition on imatinib

Chung H Kok[#,1,2,3,4], Verity A Saunders[#,1], Phuong Dang[1], Naranie Shanmuganathan[1,2,3,4,5,6,7], Deborah White[1,2,7], Susan Branford[2,3,4,6], David Yeung[*,1,2,5,7] and Timothy P Hughes[*,1,2,5,7]

Affiliations:

1. Precision Cancer Medicine Theme, South Australian Health & Medical Research Institute (SAHMRI), Adelaide, Australia
2. Adelaide Medical School, University of Adelaide, Adelaide, Australia
3. Centre for Cancer Biology, SA Pathology, Adelaide, Australia
4. Clinical Health Sciences, University of South Australia, Adelaide, Australia
5. Department of Haematology, Royal Adelaide Hospital and SA Pathology, Adelaide, Australia
6. Department of Genetics and Molecular Pathology, SA Pathology, Adelaide, Australia
7. Australasian Leukaemia and Lymphoma Group (ALLG)

#Joint first authorship

*Joint senior authorship

This file contains:

- **Supplementary Methods**
- **Supplementary Table (S1 to S3)**
- **Supplementary Figures (S1 to S14)**
- **Additional Results (SR1 to SR5)**
- **References**

Supplementary Methods

Preparation of protein lysates and western blot analysis

Mononuclear cells were isolated from blood using Lymphoprep™ (STEMCELL Technologies, Vancouver, BC). A total of $2x10^6$ mononuclear cells were lysed in 1x Laemmli's buffer with added phosphatase inhibitors, 10 mM sodium fluoride and 1 mM sodium orthovanadate, by boiling for 12 minutes. Lysates were clarified by microcentrifugation for 5 minutes at 15,000 relative centrifugal force and stored at -80°C until Western blot was performed. Twenty microliters of protein lysate (corresponding to 2 million cells) were resolved on a sodium dodecyl sulfate/14% polyacrylamide gel. Sequential samples from the same patient were run on the same gel to minimize interassay variability, alongside a negative control lysate equivalent to $2x10^5$ cells of the *BCR::ABL1*-negative HL60 cell-line. The protein was electrophoretically transferred to a polyvinylidene difluoride membrane (Amersham, Piscataway, NJ) and probed with anti-Crkl antibody (cat.no. sc-319; Santa Cruz Biotechnology, Santa Cruz, CA). Bound antibodies were detected with an enhanced chemifluorescence substrate (Amersham Pharmacia) and analyzed using the Fluor Imager (Molecular Dynamics, Sunnyvale, CA). Signals were quantified using Image Quant software (Molecular Dynamics) with HL60 no pCrkl control value subtracted from each patient %pCrkl value.

BCR::ABL1 rate of decline measurements

In this study, we used previously defined *BCR::ABL1* mRNA halving time quartiles (Q1 <9.35 days, Q2: 9.35-13.95 days, Q3: 13.96-21.85 days, and Q4: >21.85 days), which were determined using a cohort of patients treated frontline with various doses of imatinib or second generation of TKIs with vast majority of imatinib-treated patients.[1] *BCR::ABL1* halving time was calculated from available baseline (sample measured at or soon after diagnosis and before commencement of any TKI therapy) and 3-month *BCR::ABL1* values using the formula as previously described.[1]

Measurement of trough imatinib level

As previously described[2], plasma concentrations of imatinib were measured using liquid chromatography and tandem mass spectrometry with deuterated imatinib as the internal standard. Trough levels were measured from the same blood draw as the IVKI assay at day 7.

Propensity score matching

To compare outcomes for patients treated with imatinib or nilotinib as the first line, we performed the propensity score matching to account for *in vivo* kinase inhibition as covariate. Propensity score calculation was performed using "nearest neighbour" algorithm with 1:1 ratio without replacement, discarding units outside a region of common support for both nilotinib and imatinib groups, and a propensity score estimated using logistic regression of the treatment on the covariates. Nearest neighbour matching is a widely used method. A distance is computed between each treated unit and each control unit, and one by one, each treated unit is assigned a control unit as a match. The matching is "greedy" in the sense that

there is no action taken to optimize an overall criterion; each match is selected without considering the other matches that may occur subsequently. After matching, the nilotinib group had 44 patients matched, 24 were unmatched and 5 were discarded because their propensity scores fell outside the corresponding region. For the imatinib group, 44 patients were matched, 124 patients were unmatched, and 5 patients were discarded. The assessment of covariates balance was visualized by distribution of propensity score plot, and standardized mean differences plot. Additionally, Mann-Whitney test was used to assess the overall balance in the cohort before matching (p<0.001) and after matching (p=0.835; Supplementary Table 1 and Supplementary Fig 11-12). R packages used in this analysis were matchit and cobalt. All the analyses were performed using R statistical software (version 4.1.1).

Patient samples

Blood samples at diagnosis (baseline, pre-TKI therapy) and after the first 7 days of treatment were collected for IVKI assessment from adult chronic phase CML (CP-CML) patients enrolled in the Australian TIDEL-II study[3] (ACTRN12607000325404; 600mg IM; n=173), Australian patients enrolled to the Tyrosine Kinase Inhibitor Optimization and Selectivity study (TOPS[4], NCT00124748; 400 mg IM; n=8, or 800 mg IM as 400 mg BID; n=24), and the Evaluating Nilotinib Efficacy and Safety in Clinical Trials-Extending Molecular Responses (ENESTxtnd[5], NCT01254188; 300 mg BID nilotinib; n=73) study.

Model selection by Akaike Information Criterion

We have used the model selection by Akaike Information Criterion (AIC) to select a multivariate model of best fit to predict EMR failure. Using the standard statistical practice, the stepwise selection (combination of forward and backward selection) which uses a strategy of stepwise regression to find a subset of variables resulting in the best performing model.[6, 7] It starts with no predictors, then sequentially adds the most predictive variables (forward selection). After adding each new variable, it removes any variables that no longer provide further improvement in the model (backward selection). The variables used in this analysis were IVKI, spleen size, IVKI + spleen size (combined), age, platelets count, blasts percentage, gender, transcript type, imatinib plasma trough level at day 7, Sokal and ELTS. As a result, the model with only IVKI + spleen size (combined) was identified as the best performing model (OR 11.4, 95% CI: 3.1-44.2, p=0.0002) compared to all other combinations tested. Accordingly, this is what we are now reporting.

Supplementary Table

Supplementary Table 1. Comparison of performance metrics of IVKI and spleen size with current prognostic scoring systems that include spleen size in predicting EMR failure.

Metrics (EMR failure)	IVKI + Spleen	Sokal	ELTS	EUTOS
Accuracy	**0.88**	0.78	0.83	0.79
Kappa	**0.32**	0.13	0.13	0.14
Sensitivity	0.29	**0.32**	0.21	0.29
Specificity	**0.97**	0.84	0.91	0.86
PosPredValue	**0.55**	0.21	0.24	0.23
NegPredValue	**0.91**	0.90	0.90	0.89
Precision	**0.55**	0.21	0.24	0.23
Recall	0.29	**0.32**	0.21	0.29
F1	**0.38**	0.25	0.22	0.26

***all values ranged from 0 to 1. The higher the score the better performance.**

Supplementary Table 2. Multivariate analysis of lVKI + spleen size and other clinical variables to predict EMR failure.

Variable	adjusted odd ratio	95% CI lower	95% CI upper	p.value
**large spleen + low IVKI vs others**	**9.41**	**2.14**	**44.16**	**0.003**
Age (years)	1.01	0.97	1.04	0.727
Platelets count	1.00	1.00	1.00	0.313
Blasts %	1.09	0.86	1.33	0.451
Gender: Male vs Female	1.30	0.42	4.29	0.655
Transcript type: b2a2 vs b3a2	2.11	0.64	7.68	0.232
IM plasma trough level on day 7	1.00	1.00	1.00	0.137

*IM = imatinib

Variable	adjusted odd ratio	95% CI lower	95% CI upper	p.value
**large spleen + low IVKI vs others**	**13.43**	**2.92**	**74.03**	**0.001**
High Sokal vs int/low Sokal	0.91	0.18	3.38	0.903

Variable	adjusted odd ratio	95% CI lower	95% CI upper	p.value
**large spleen + low IVKI vs others**	**30.55**	**4.26**	**620.56**	**0.003**
High ELTS vs int/low ELTS	0.29	0.01	2.17	0.311

Variable	adjusted odd ratio	95% CI lower	95% CI upper	p.value
**large spleen + low IVKI vs others**	**23.49**	**3.22**	**484.47**	**0.007**
High EUTOS vs int/low EUTOS	0.42	0.02	2.42	0.428

Supplementary Table 3. The level of IVKI in both imatinib and nilotinib groups before and after adjusted by propensity score matching.

Before adjusted	imatinib	nilotinib	p
n	173	73	
IVKI (median [IQR])	23.30 [11.87, 38.76]	56.43 [41.02, 66.63]	<0.001
**After adjusted**			
n	44	44	
IVKI (median [IQR])	45.08 [33.81, 54.44]	44.83 [33.79, 56.42]	0.835

IQR = interquartile range

Supplementary Figures

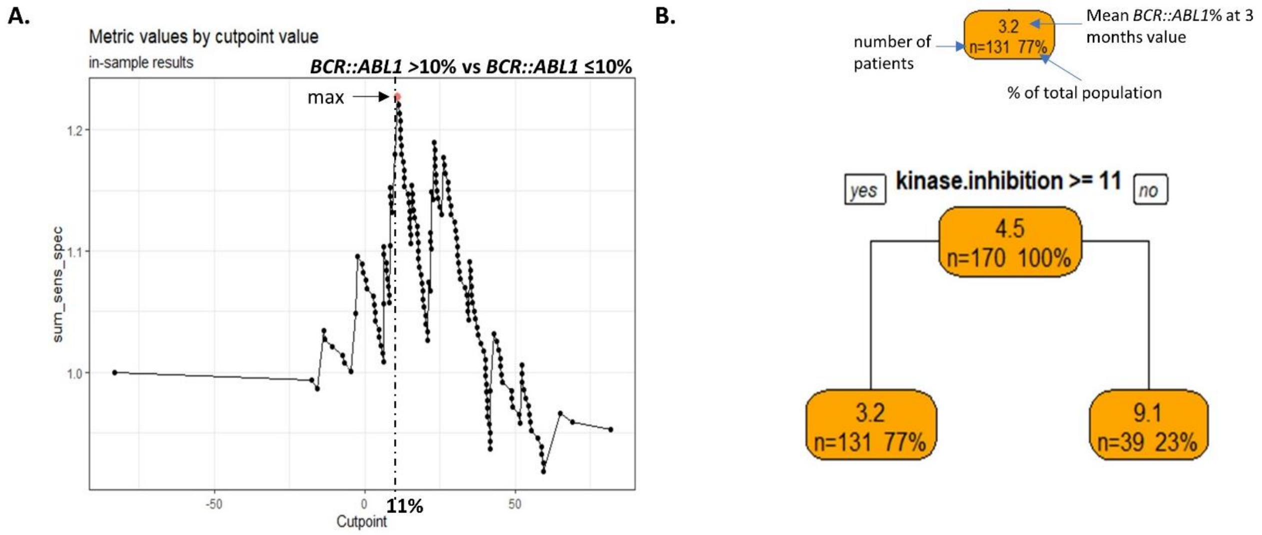

Supplementary Fig 1. Two independent statistical approaches were used to identify a threshold value for optimal kinase inhibition after the first 7 days imatinib treatment for EMR failure at 3 months. A) Line plots shows the sum of sensitivity and specificity against a range of IVKI level. Optimal cutoff for *BCR::ABL1* >10% was identified based on max sum of sensitivity and specificity. **B)** Decision tree analysis reveals IVKI ≥11% as optimal cutoff for *BCR::ABL1*% RNA level at 3 months as continuous variable. The top value in each box represents the average *BCR::ABL1%* level. For example, patients with IVKI ≥11% had average of *BCR::ABL1* 3.2% (n=131) which represents 77% of this imatinib treated patients cohort.

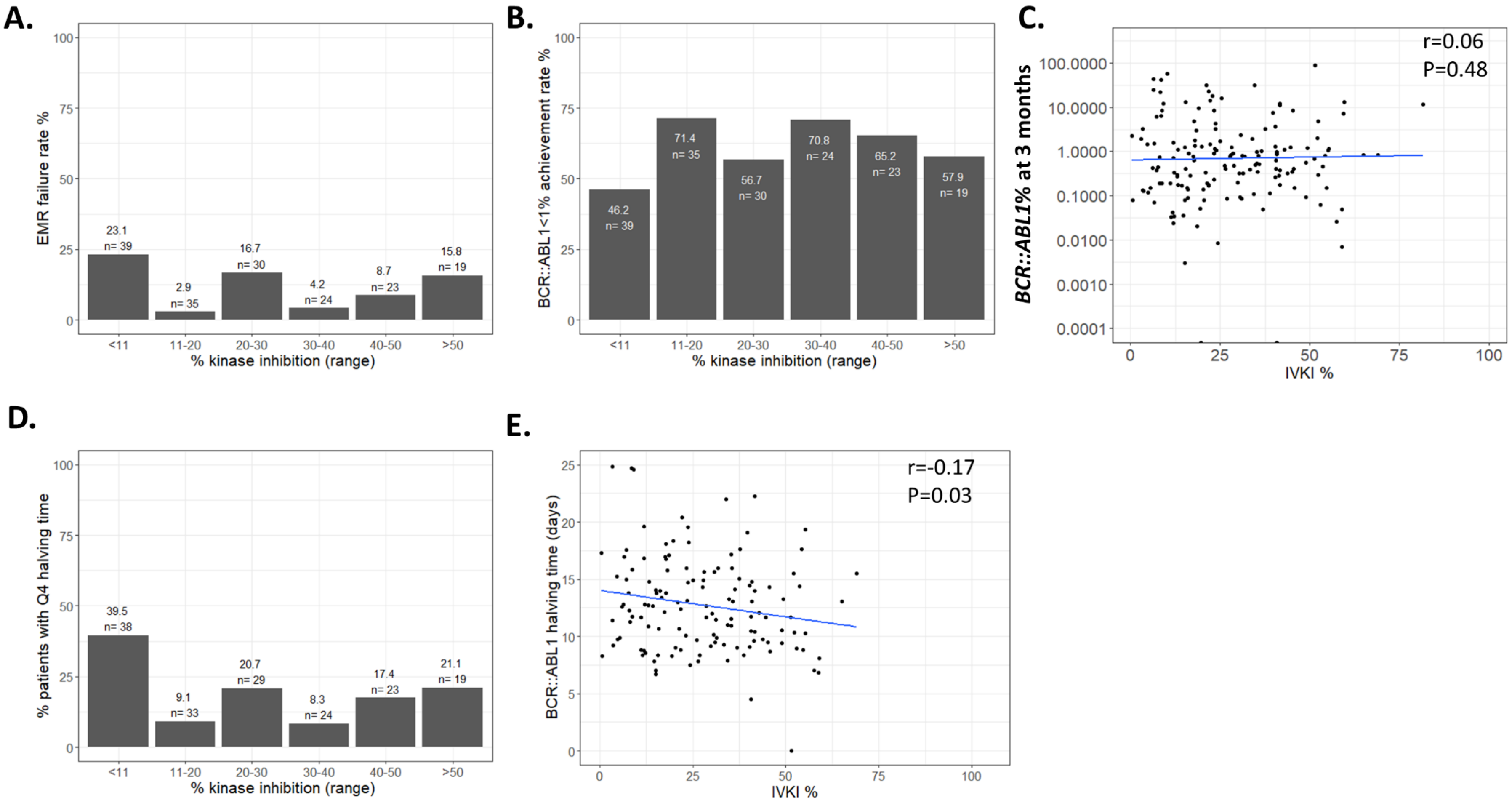

Supplementary Fig 2. The comparison of IVKI levels against molecular responses and halving time in imatinib cohort. A) Barplot shows the proportion of EMR failure patients across a range of IVKI levels in imatinib cohort. **B)** Barplot shows the proportion of patients who achieved *BCR::ABL1*≤1% at 3 months across a range of IVKI levels. **C)** The correlation between *BCR::ABL1*% at 3 months and IVKI levels. **D)** Barplot shows the proportion of patients with Q4 *BCR::ABL1* halving time across a range of IVKI levels. **E)** The correlation between the halving time (days) and IVKI levels.

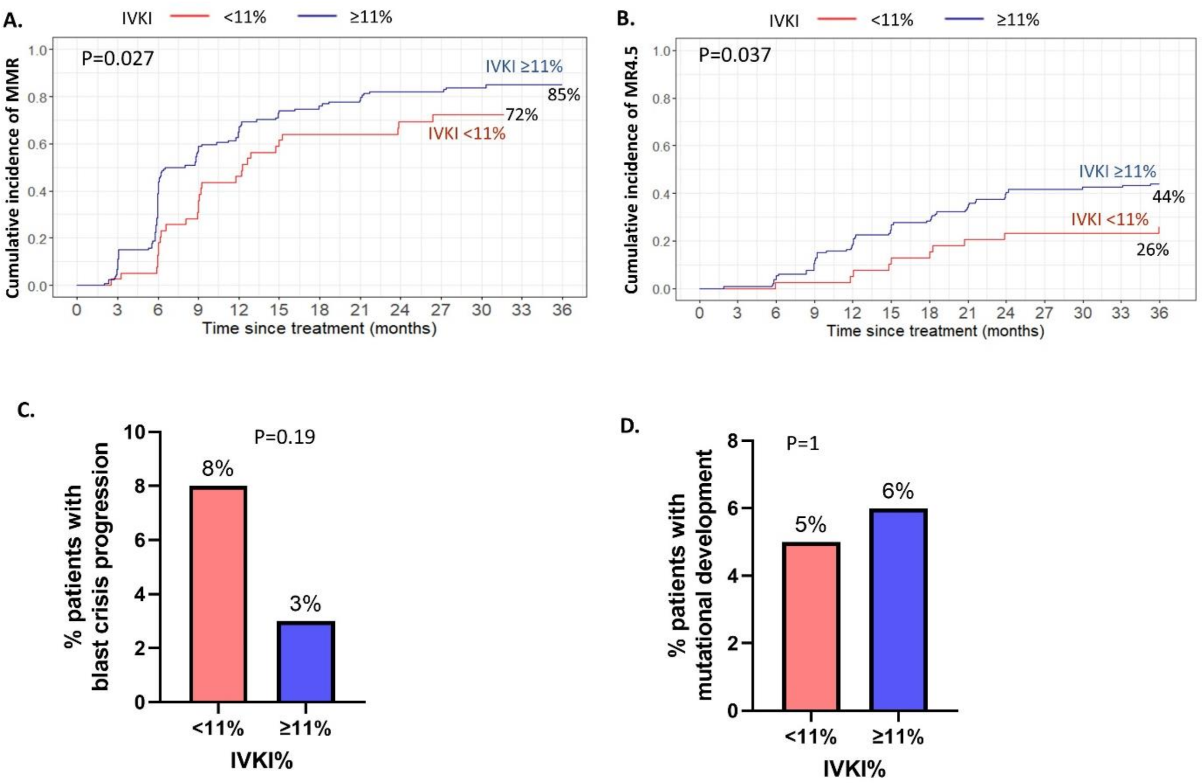

Supplementary Fig 3. The impact of *in vivo* kinase inhibition threshold during the first 7 days on molecular responses and outcome in CML patients treated with imatinib. The cumulative incidence of achieving **A)** MMR, and **B)** MR4.5 by 36 months in patients treated with imatinib stratified by low and high IVKI groups. Barplot shows the proportion of patients with **C)** blast crisis progression and **D)** *BCR::ABL1* kinase domain mutations in low and high IVKI groups.

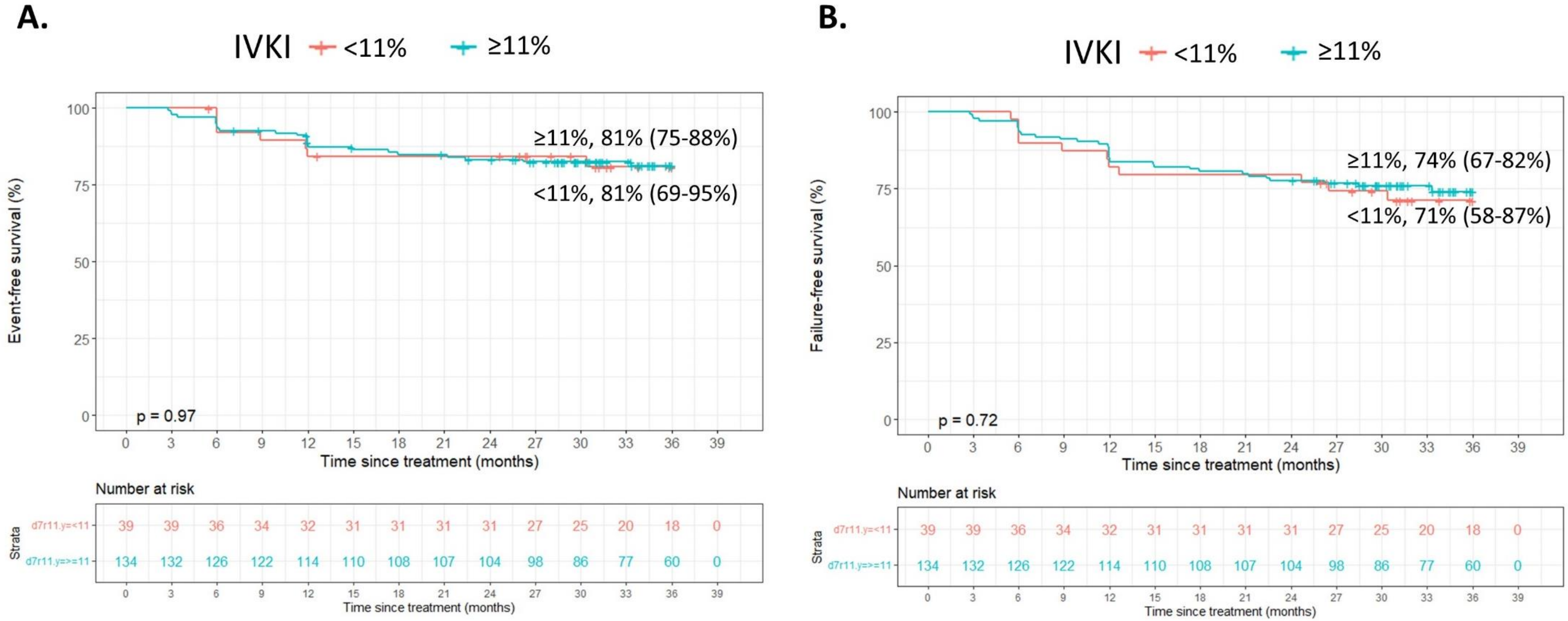

Supplementary Fig 4. No significant association between IVKI and event-free survival (EFS) or failure-free survival (FFS). The EFS (**A.**) and FFS (**B.**) in patients treated with imatinib stratified by IVKI based on 11% cutoff.

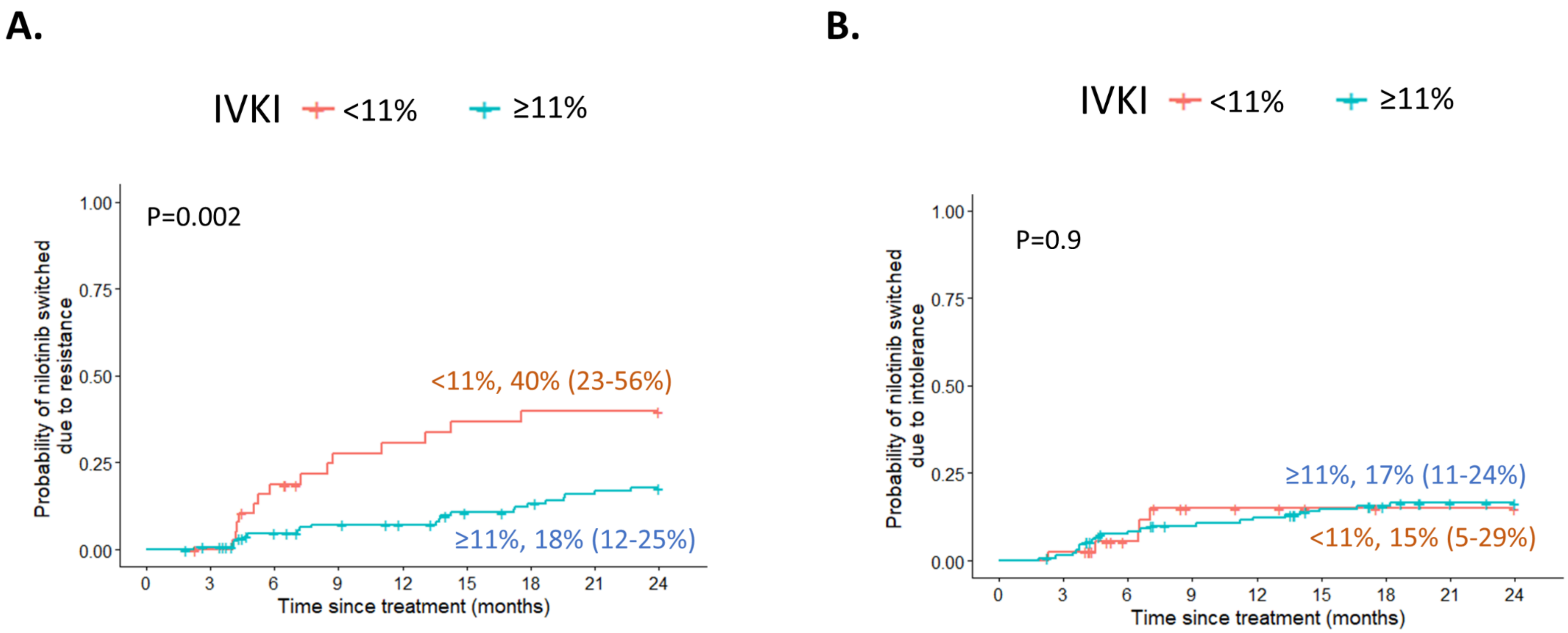

Supplementary Fig 5. Low IVKI patients had higher probability of nilotinib switched due to failure. Kaplan-Meier graph shows the estimated probability of nilotinib switched due to (**A**.) resistance/failure or (**B**.) intolerance in patients treated with imatinib stratified by IVKI threshold (11%). The p value was derived from log-rank test.

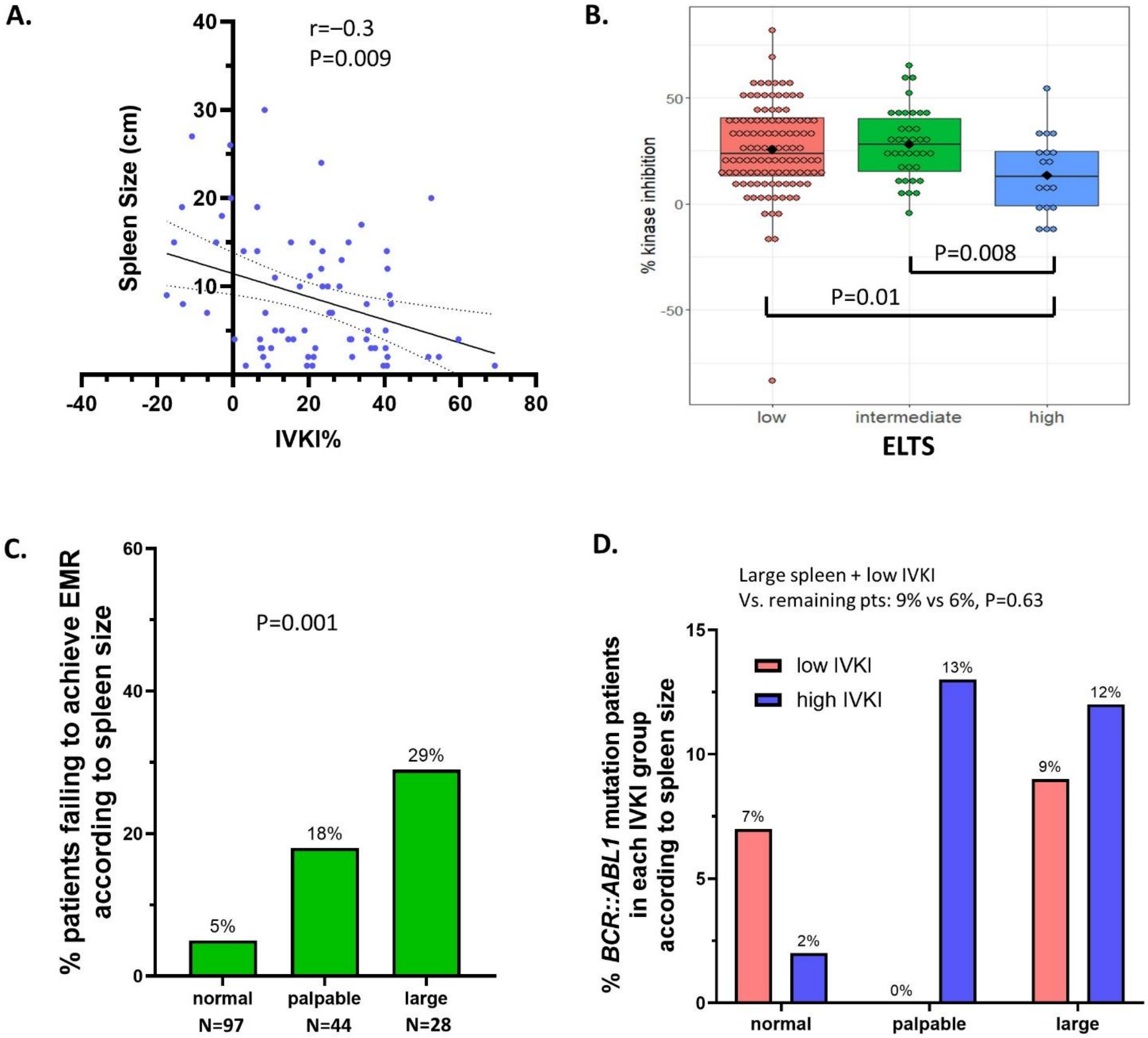

Supplementary Fig 6. Imatinib treated patients with large spleen and low IVKI had inferior early molecular response (EMR) and higher incidence of blast crisis progression. A) The correlation between spleen size and IVKI. Each dot represents a patient. **B)** Boxplot shows the IVKI level in each ELTS group. Diamond shape represents mean and horizontal line in each boxplot represents median. Each dot represents a patient sample. **C)** Barplot shows the percentage of patients failing to achieve EMR in each spleen size group *i.e.* no enlarged spleen size (normal; 0 cm), palpable spleen size (1-9 cm), and large spleen size (≥10 cm). **D)** Barplot shows the percentage of patients with mutation development according to the combination of spleen size and IVKI groups.

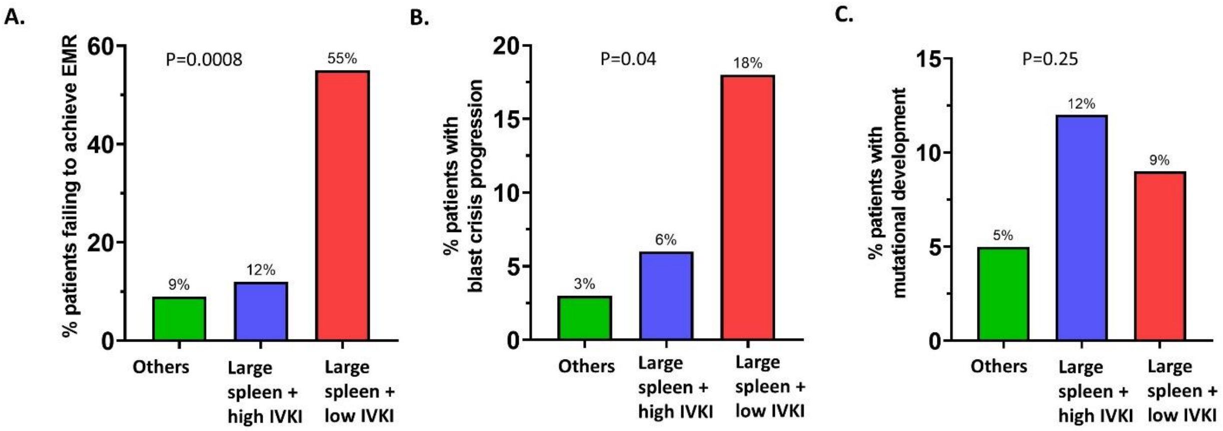

Supplementary Fig 7. Combination of large spleen and low *in vivo* kinase inhibition is an early predictor of inferior molecular response in patients treated with imatinib. A) Barplot shows the percentage of patients failing to achieve EMR according to the spleen size and IVKI groups i.e. large spleen size and high IVKI (large spleen + high IVKI), large spleen size and low IVKI (large spleen + low IVKI), and the remaining patients (Others). **B)** Barplot shows the percentage of blast crisis progression patients according to the combination of spleen size and IVKI groups. **C)** Barplot shows the percentage of patients with BCR::ABL1 kinase domain mutation development according to the combination of spleen size and IVKI groups.

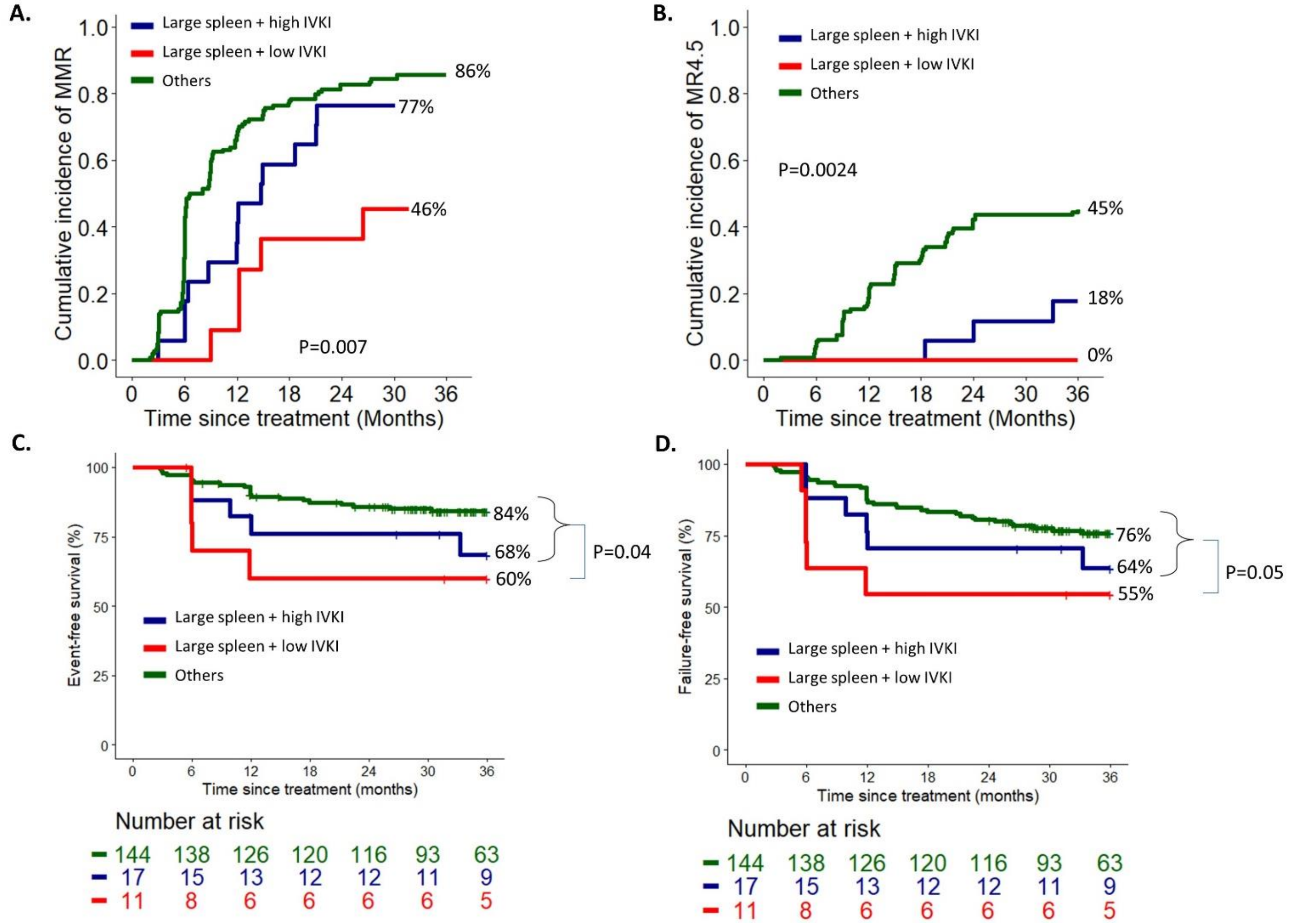

Supplementary Fig 8. Imatinib treated patients with large spleen and low IVKI had inferior molecular responses and outcome. The cumulative incidence of achieving (**A.**) MMR, and (**B.**) MR4.5 by 36 months in patients treated with imatinib stratified by the combination of spleen size and IVKI groups. The event-free survival (EFS) (**C.**) and failure-free survival (FFS) (**D.**) in patients treated with imatinib stratified by the combination of spleen size and IVKI groups i.e. large spleen size and high IVKI (large spleen size + high IVKI), large spleen size and low IVKI (large spleen + low IVKI), and the remaining patients (Others).

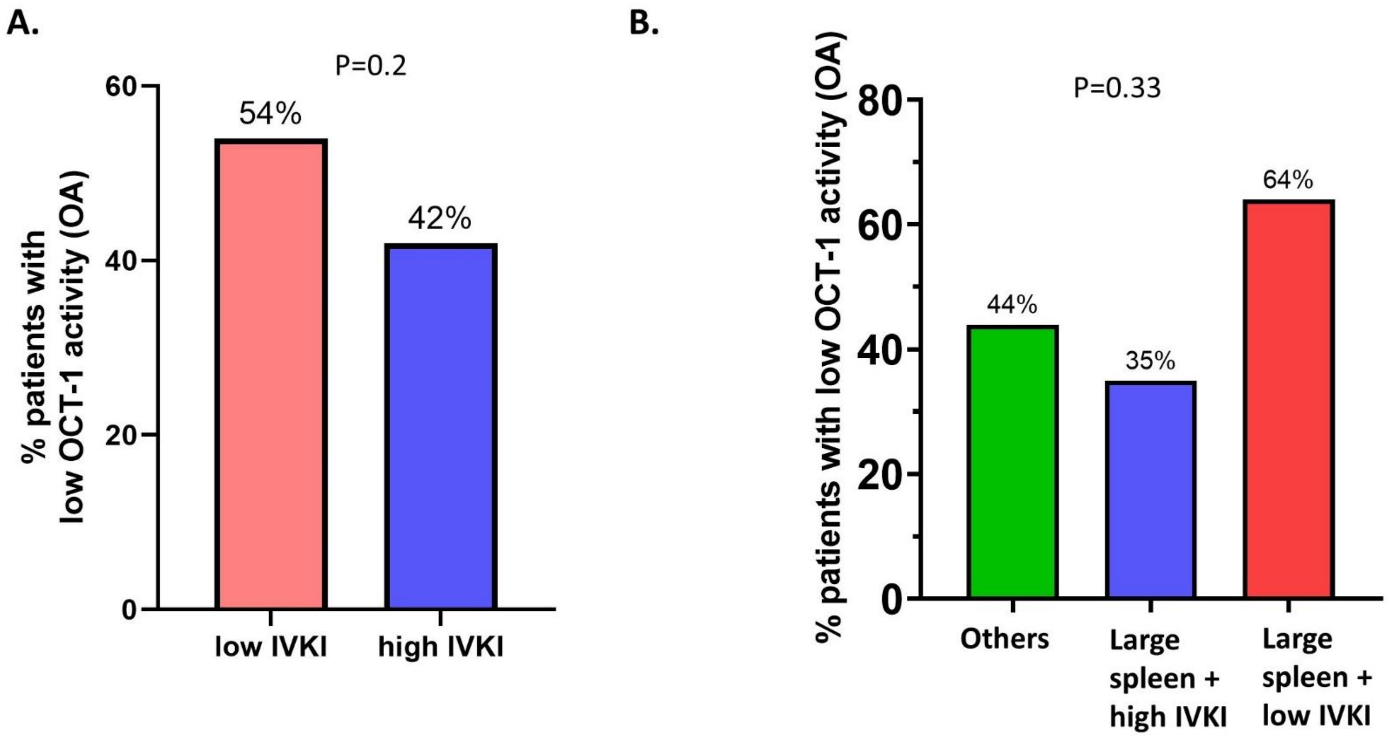

Supplementary Fig 9. The relationship between OCT-1 activity and *in vivo* kinase inhibition in patients treated with imatinib. A) Barplot shows the percentage of patients with low OCT-1 activity (OA) according to IVKI. Low OA defined as imatinib uptake <4ng/100,000 cells. **B)** Barplot shows the percentage of patients with low OA according to the spleen size and IVKI groups i.e. large spleen size and high IVKI (large spleen size + high IVKI), large spleen size and low IVKI (large spleen + low IVKI), and the remaining patients (Others).

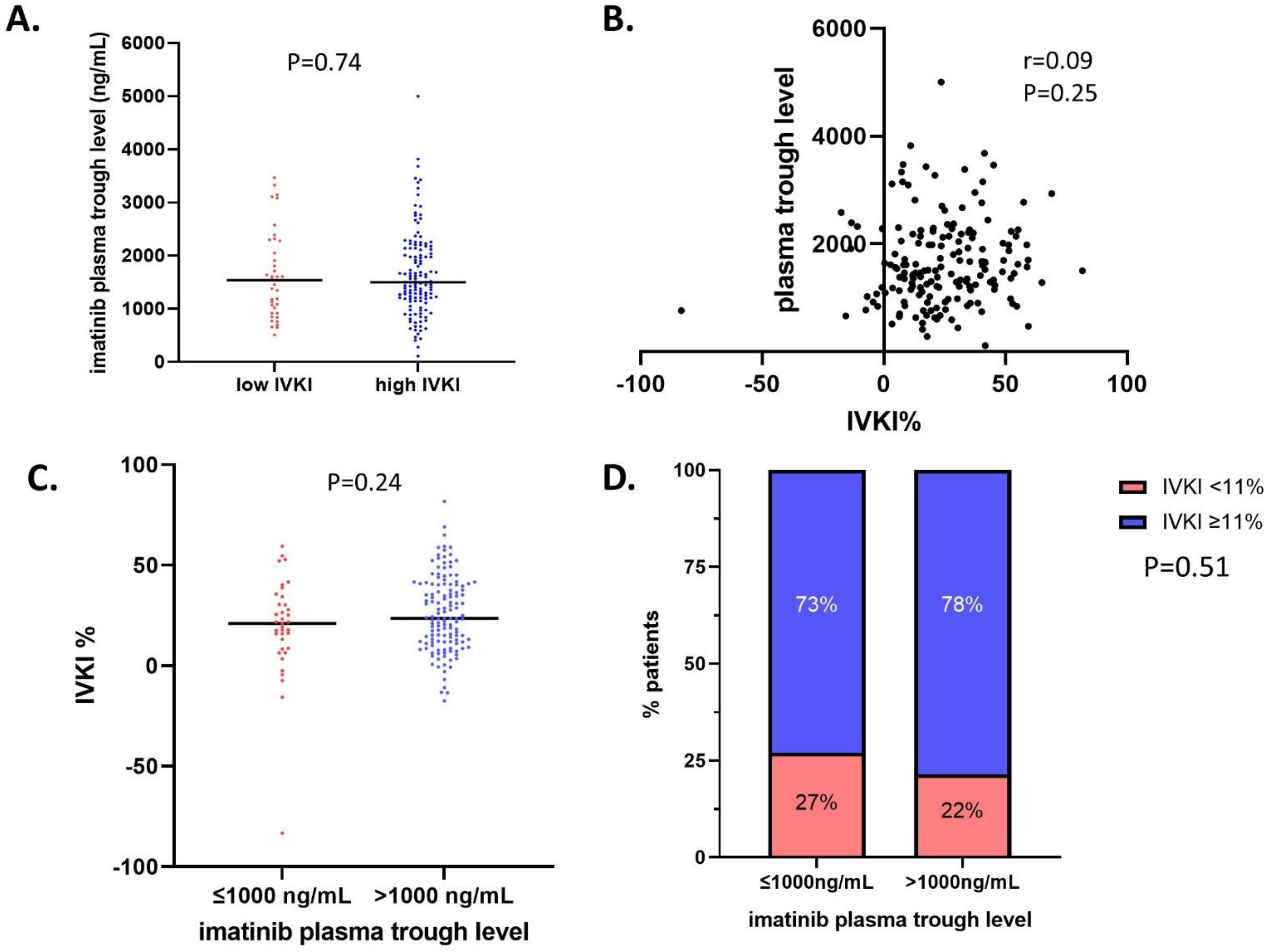

Supplementary Fig 10. No correlation between imatinib plasma trough level and IVKI. A) Dotplot shows the imatinib plasma trough level measured at day 7 between low and high IVKI. The horizontal bar indicates median. **B)** The correlation between imatinib plasma trough level and IVKI at day 7. **C)** Dotplot shows the level of IVKI between high (>1000 ng/mL) and low (≤1000 ng/mL) imatinib plasma trough level measured at day 7. The horizontal bar indicates median. **D)** Barplot shows the proportion of low and high IVKI patients between high and low imatinib plasma trough level.

***BCR::ABL1* halving time**

	Q1	Q2	Q3	Q4
nilotinib	37.5	46.9	10.9	4.7
imatinib	18.7	34.3	26.5	20.5

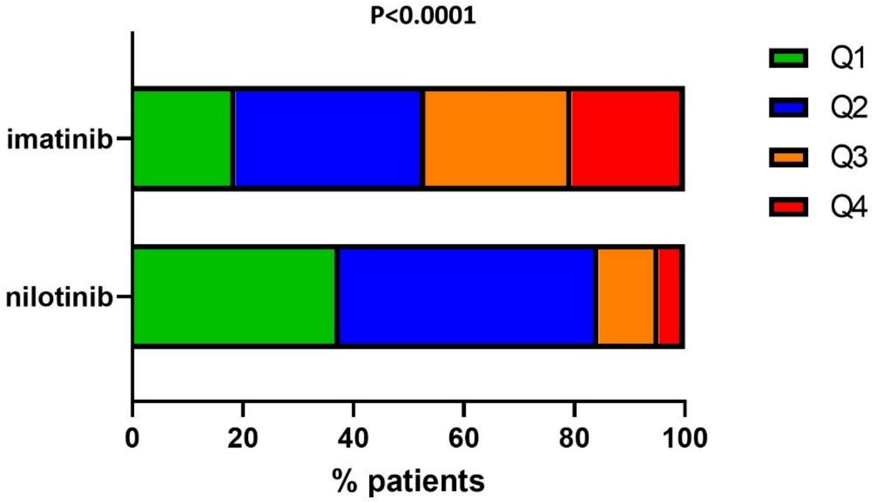

Supplementary Fig 11. The comparison of *BCR::ABL1* halving time quartiles between imatinib and nilotinib treated patients. The table (top) shows the proportion in percentage of *BCR::ABL1* halving time quartiles across each TKI. Barplot (bottom) shows the proportion of *BCR::ABL1* halving time quartiles for each TKI. For the halving time quartile, Q1 < 9.35 days, Q2: 9.35-13.95 days, Q3: 13.96-21.85 days, and Q4: >21.85 days. Q4 defined as the longest halving time and Q1 defined as the shortest halving time.

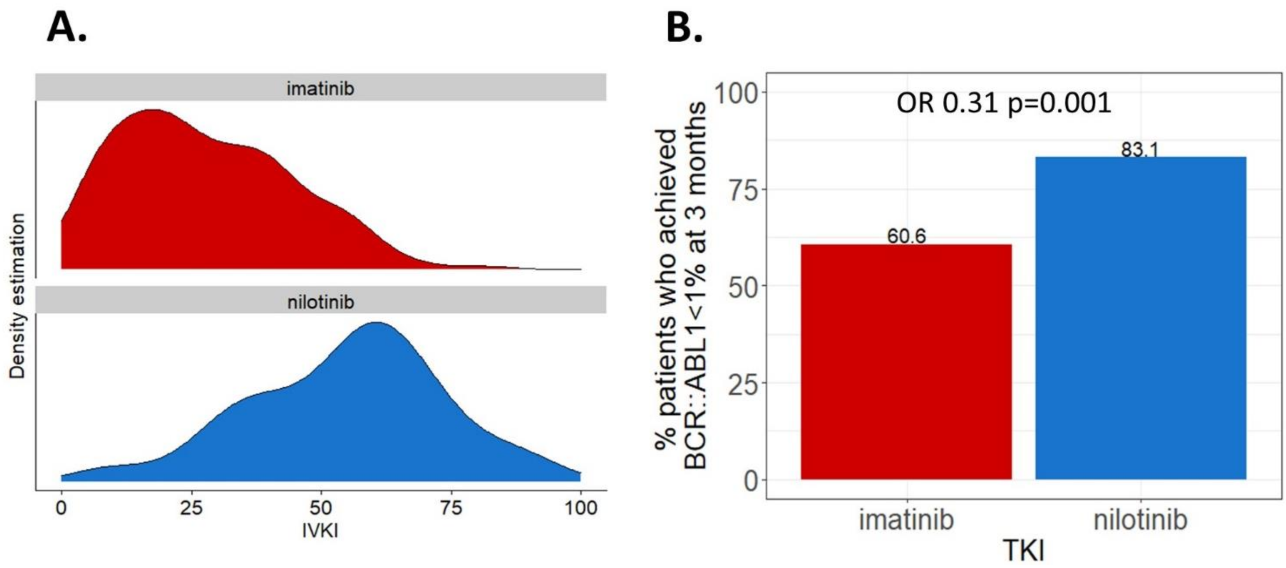

Supplementary Fig 12. Comparison of achievement of *BCR::ABL1<1%* at 3 months between imatinib treated or nilotinib treated patients. A) Density plot shows the comparison of IVKI % distribution in imatinib and nilotinib cohorts. **B)** Barplot shows the proportion of patients achieving *BCR::ABL1* ≤1% at 3 months in imatinib and nilotinib cohorts.

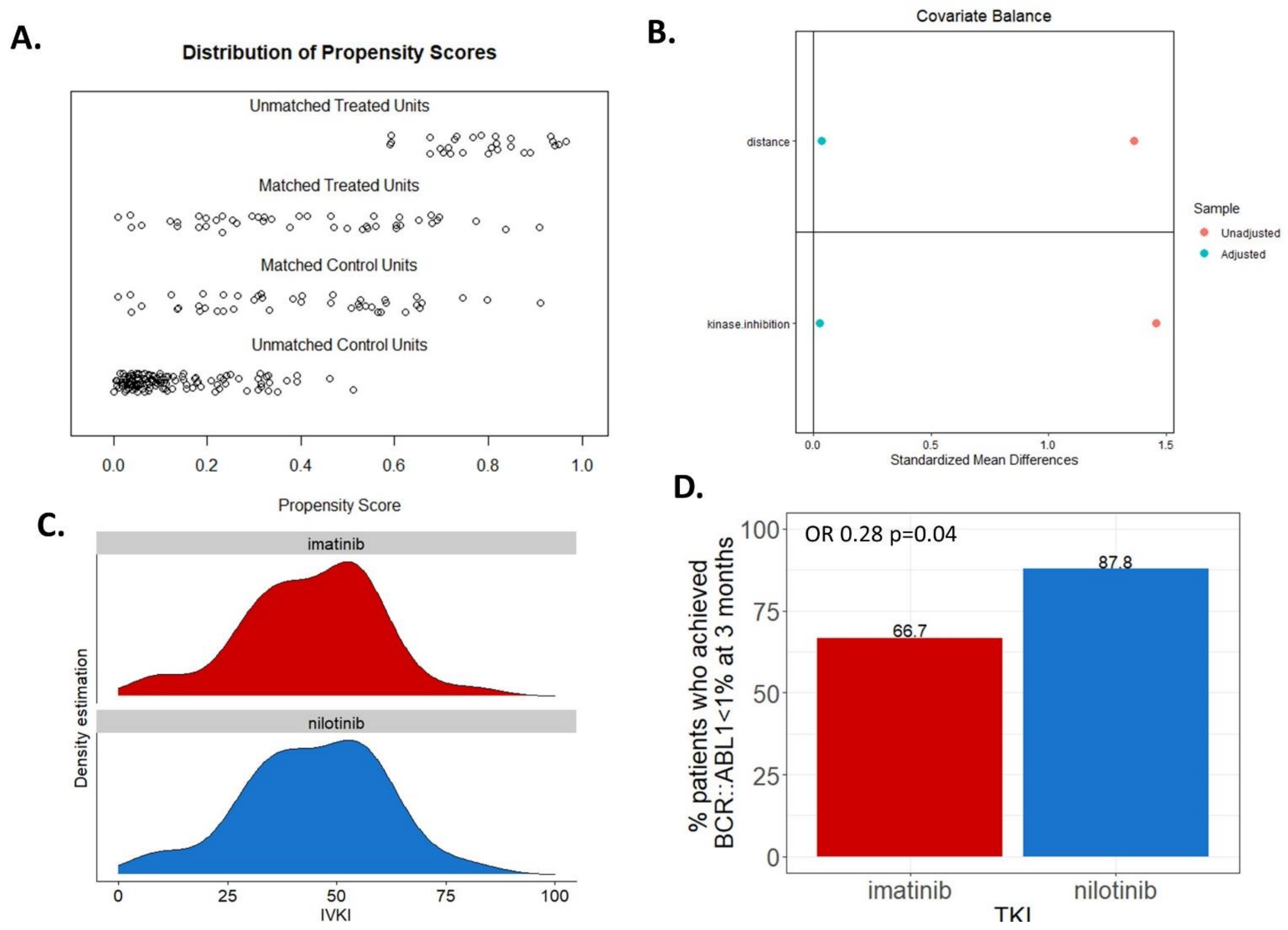

Supplementary Fig 13. Propensity score matching analysis based on TKI as dependent variable and IVKI as covariate. A) Distribution of propensity scores for unmatched and matched control (imatinib) and treated (nilotinib) cohorts. **B)** The covariate balance plot shows that after adjusted for IVKI (cyan) the standardized mean differences was closer to 0 indicates covariate balanced compared to unadjusted IVKI (red). **C)** Density plot shows the comparison of IVKI % distribution in imatinib and nilotinib cohorts after adjustment of IVKI by propensity matching analysis. **D)** Barplot shows the proportion of patients achieving *BCR::ABL1* ≤1% at 3 months in imatinib and nilotinib cohorts after adjustment of IVKI by propensity matching analysis.

A.

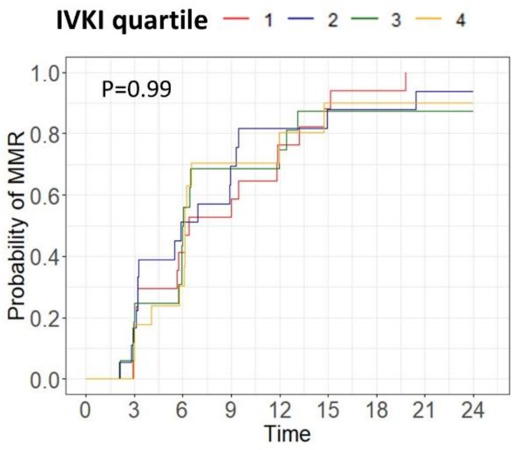

Time (months)	Cumulative incidence of MMR	
	group	estimate (CI)
24 months	1	0.941 (0.462,0.995
	2	0.939 (0.449,0.995)
	3	0.875 (0.516,0.973)
	4	0.901 (0.291,0.991)

B.

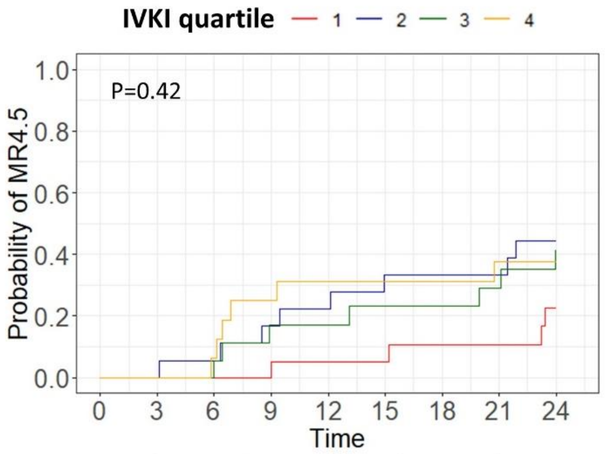

Time (months)	Cumulative incidence of MR4.5	
	group	estimate (CI)
24 months	1	0.227 (0.066,0.447)
	2	0.444 (0.207,0.659)
	3	0.412 (0.175,0.637)
	4	0.375 (0.145,0.607)

Supplementary Fig 14. No difference was identified between IVKI and molecular responses in patients treated with nilotinib. The IVKI was divided into quartiles to determine the impact of IVKI quartile on molecular response by 24 months in nilotinib treated cohort (ENESTxtnd)[5]. The cumulative incidence of **A)** MMR and **B)** MR4.5 stratified by IVKI quartiles defined as Q1 < 41%, Q2: 41-56.4%, Q3: 56.5-66.6%, and Q4 >66.6%.

Additional Results

Reproducibility of pCrkl kinase assay

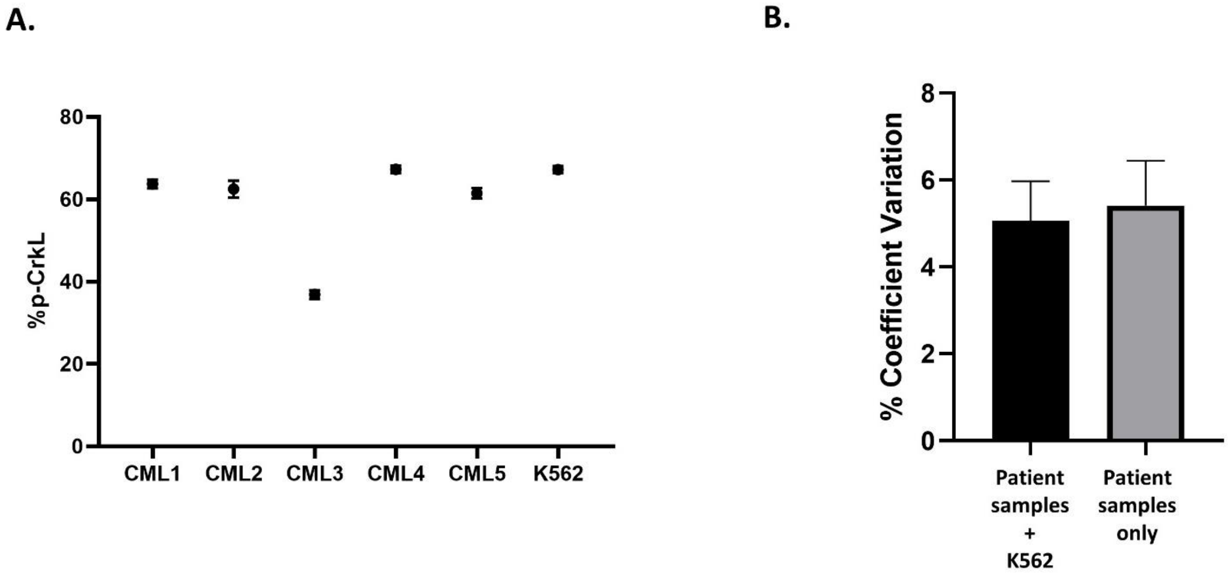

Supplementary Fig SR1. The technical reproducibility of p-Crkl kinase assay. A) The %p-Crkl for each independent CML patient and K562 cell line repeated at least 5 times and by different operators. Error bars represent standard error measurement. **B**) The barplot shows the average coefficient variation for 5 CML patient samples at diagnosis, untreated, with or without K562 cell line comparison, where %p-Crkl protein level was assessed at least 5 times in independent Western blot assays. Error bars represent standard error measurement.

We used the coefficient of variation (CV) to determine the technical reproducibility of this western-blot based assay by using the K562 cell line and 5 independent CML patient samples with at least 5 independent replicates, and by different operators. We calculated that this assay on average had 5.4% CV (range: 3.2-9.1%, Supplementary Figure SR1).

No correlation between pCrkl at baseline and *BCR::ABL1%* IS transcript level at baseline

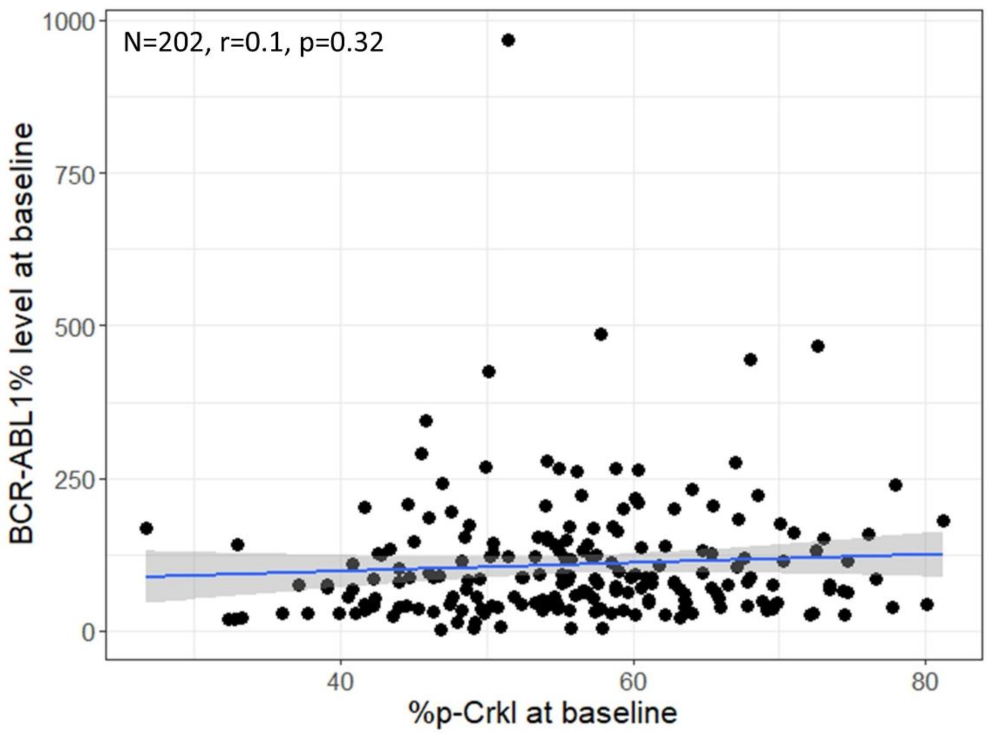

Supplementary Fig SR2. No correlation between baseline kinase activity and the RNA level of *BCR::ABL1%* at baseline.

There was no correlation between pCrkl level at baseline and *BCR::ABL1%* IS transcript level at baseline (r=0.1, p=0.32; Supplementary Figure SR2).

The relationship between no kinase inhibition and trough imatinib plasma level at day 7

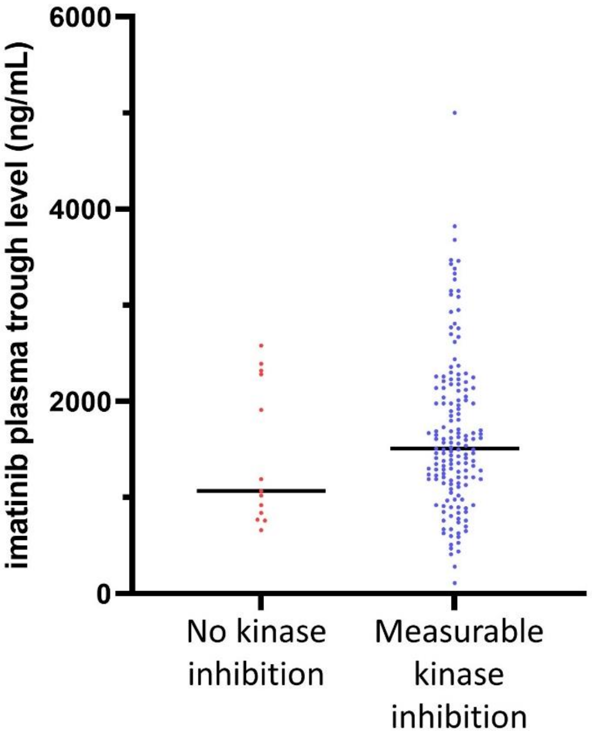

Supplementary Fig SR3. The relationship between no kinase inhibition and trough imatinib plasma level at day 7. The dotplot shows the imatinib plasma trough level measured at day 7 between no kinase inhibition (IVKI ≤0) and measurable kinase inhibition (IVKI >0). The horizontal bar indicates median.

There were 13 (8%) patients who did not show inhibition of kinase *in vivo* on day 7. Of these 13 patients, 9 (69%) patients subsequently failed to reach TIDEL-II defined molecular milestones. These 13 patients had similar imatinib plasma trough level at day 7 to those patients with measurable kinase inhibition (1070 ng/mL vs 1510 ng/mL, p=0.31, Supplementary Figure SR3). Thus, we could confirm that these patients had taken their TKI the day before blood collection and measurement.

Optimal IVKI threshold level for determining halving time was 11%

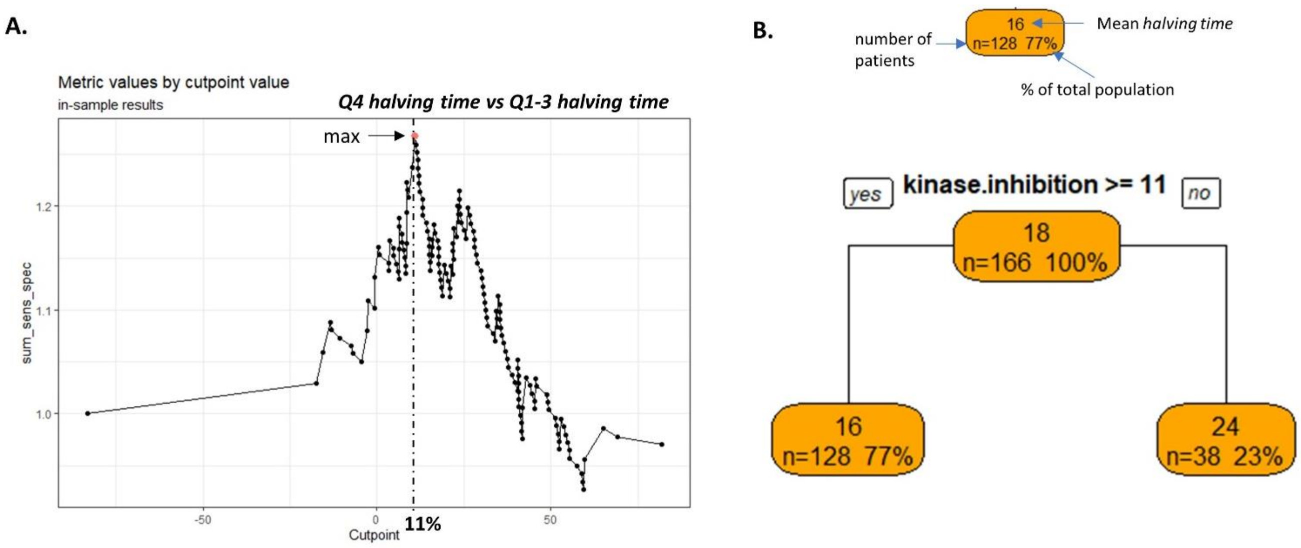

Supplementary Fig SR4. Two independent statistical approaches were used to identify a threshold value for optimal kinase inhibition after the first 7 days imatinib treatment for *BCR::ABL1 halving time*. A) Line plots shows the sum of sensitivity and specificity against a range of IVKI level. Optimal cutoff for Q4 *BCR::ABL1* halving time was identified based on max sum of sensitivity and specificity. **B)** Decision tree analysis reveals IVKI ≥11% as optimal cutoff for *BCR::ABL1 halving time* as continuous variable. The top value in each box represents the average *BCR::ABL1 halving time*. For example, patients with IVKI ≥11% had average of *BCR::ABL1 halving time 16 days* (n=128) which represents 77% of this imatinib treated patients cohort.

We also identified the optimal IVKI threshold level for determining halving time was 11% (Supplementary Figure SR4), which is the same cutoff level determined for EMR failure.

Optimal IVKI threshold level for determining *BCR::ABL1* ≤1% was 11%

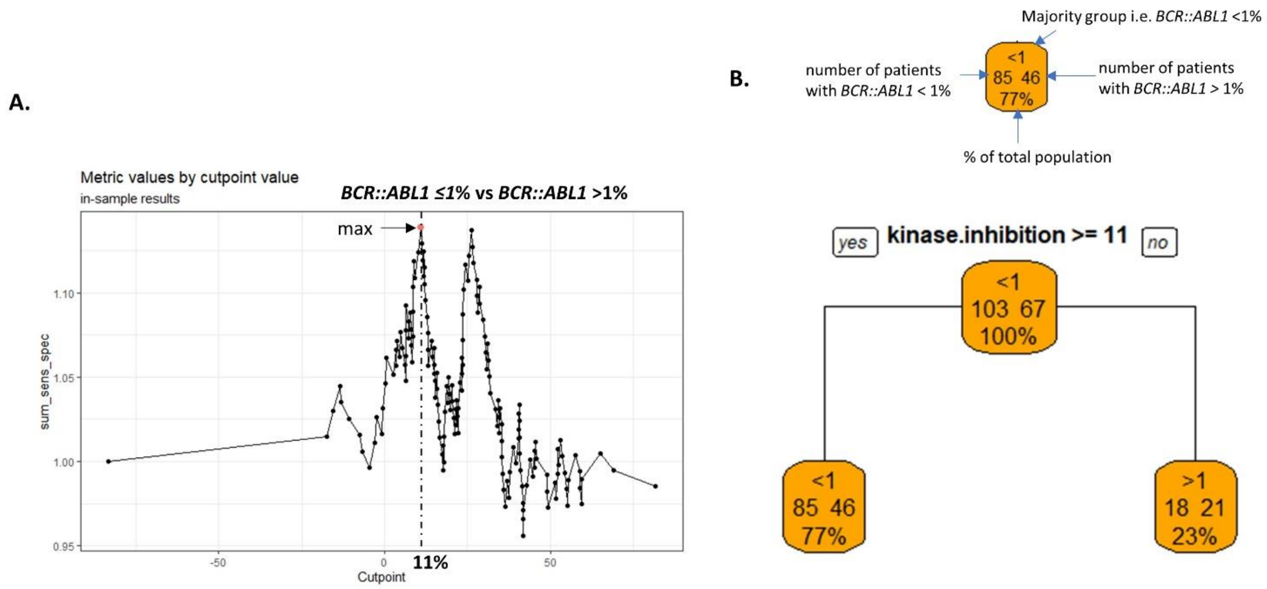

Supplementary Fig SR5. Two independent statistical approaches were used to identify a threshold value for optimal kinase inhibition after the first 7 days imatinib for *BCR::ABL1* ≤1% RNA level at 3 months. A) Line plots shows the sum of sensitivity and specificity against a range of IVKI level. Optimal cutoff for *BCR::ABL1* ≤1% was identified based on max sum of sensitivity and specificity. **B)** Decision tree analysis reveals IVKI ≥11% as optimal cutoff for *BCR::ABL1* ≤1% RNA level at 3 months. The middle value in each box represents total patients of *BCR::ABL1* ≤1% (left) and *BCR::ABL1*>1% (right). For example, patients with IVKI ≥11% was overrepresented by *BCR::ABL1* ≤1% (n=85) and *BCR::ABL1* >1% (n=46) which represents 77% of this imatinib treated patients cohort.

We also identified the optimal IVKI threshold level for determining *BCR::ABL1* ≤1% was 11% (Supplementary Figure SR5), which is the same cutoff level determined for EMR failure.

Taken together, 11% IVKI was consistently identified as a threshold for achieving optimal halving time and low *BCR::ABL1* level at 3 months.

References

1. Shanmuganathan N, Pagani IS, Ross DM, Park S, Yong ASM, Braley JA*, et al.* Early BCR-ABL1 kinetics are predictive of subsequent achievement of treatment-free remission in chronic myeloid leukemia. *Blood* 2021 Mar 4; **137**(9)**:** 1196-1207.

2. White DL, Radich J, Soverini S, Saunders VA, Frede AK, Dang P*, et al.* Chronic phase chronic myeloid leukemia patients with low OCT-1 activity randomized to high-dose imatinib achieve better responses and have lower failure rates than those randomized to standard-dose imatinib. *Haematologica* 2012 Jun; **97**(6)**:** 907-914.

3. Yeung DT, Osborn MP, White DL, Branford S, Braley J, Herschtal A*, et al.* TIDEL-II: frontline use of imatinib in CML with early switch to nilotinib for failure to achieve time-dependent molecular targets. *Blood* 2014 Dec 17.

4. Cortes JE, Baccarani M, Guilhot F, Druker BJ, Branford S, Kim DW*, et al.* Phase III, randomized, open-label study of daily imatinib mesylate 400 mg versus 800 mg in patients with newly diagnosed, previously untreated chronic myeloid leukemia in chronic phase using molecular end points: tyrosine kinase inhibitor optimization and selectivity study. *Journal of clinical oncology : official journal of the American Society of Clinical Oncology* 2010 Jan 20; **28**(3)**:** 424-430.

5. Hughes TP, Munhoz E, Aurelio Salvino M, Ong TC, Elhaddad A, Shortt J*, et al.* Nilotinib dose-optimization in newly diagnosed chronic myeloid leukaemia in chronic phase: final results from ENESTxtnd. *British journal of haematology* 2017 Oct; **179**(2)**:** 219-228.

6. Bruce P, Bruce A. *Practical Statistics for Data Scientists*. O'Reilly Media, 2017.

7. James G, Witten D, Hastie T, Tibshirani R. *An Introduction to Statistical Learning: With Applications in R*, Second edn. Springer Publishing Company, 2014, 615pp.
